# Origins of strabismus and loss of binocular vision

**DOI:** 10.3389/fnint.2014.00071

**Published:** 2014-09-25

**Authors:** Emmanuel Bui Quoc, Chantal Milleret

**Affiliations:** ^1^Ophthalmology Department, Hopital Robert Debre/Assistance Publique Hopitaux de ParisParis, France; ^2^Collège de France, Center for Interdisciplinary Research in Biology (CIRB), Spatial Navigation and Memory TeamParis, France

**Keywords:** children, early strabismus, binocular vision, brain development, critical period

## Abstract

Strabismus is a frequent ocular disorder that develops early in life in humans. As a general rule, it is characterized by a misalignment of the visual axes which most often appears during the critical period of visual development. However other characteristics of strabismus may vary greatly among subjects, for example, being convergent or divergent, horizontal or vertical, with variable angles of deviation. Binocular vision may also vary greatly. Our main goal here is to develop the idea that such “polymorphy” reflects a wide variety in the possible origins of strabismus. We propose that strabismus must be considered as possibly resulting from abnormal genetic and/or acquired factors, anatomical and/or functional abnormalities, in the sensory and/or the motor systems, both peripherally and/or in the brain itself. We shall particularly develop the possible “central” origins of strabismus. Indeed, we are convinced that it is time now to open this “black box” in order to move forward. All of this will be developed on the basis of both presently available data in literature (including most recent data) and our own experience. Both data in biology and medicine will be referred to. Our conclusions will hopefully help ophthalmologists to better understand strabismus and to develop new therapeutic strategies in the future. Presently, physicians eliminate or limit the negative effects of such pathology both on the development of the visual system and visual perception through the use of optical correction and, in some cases, extraocular muscle surgery. To better circumscribe the problem of the origins of strabismus, including at a cerebral level, may improve its management, in particular with respect to binocular vision, through innovating tools by treating the pathology at the source.

## Introduction

**Figure 1:**
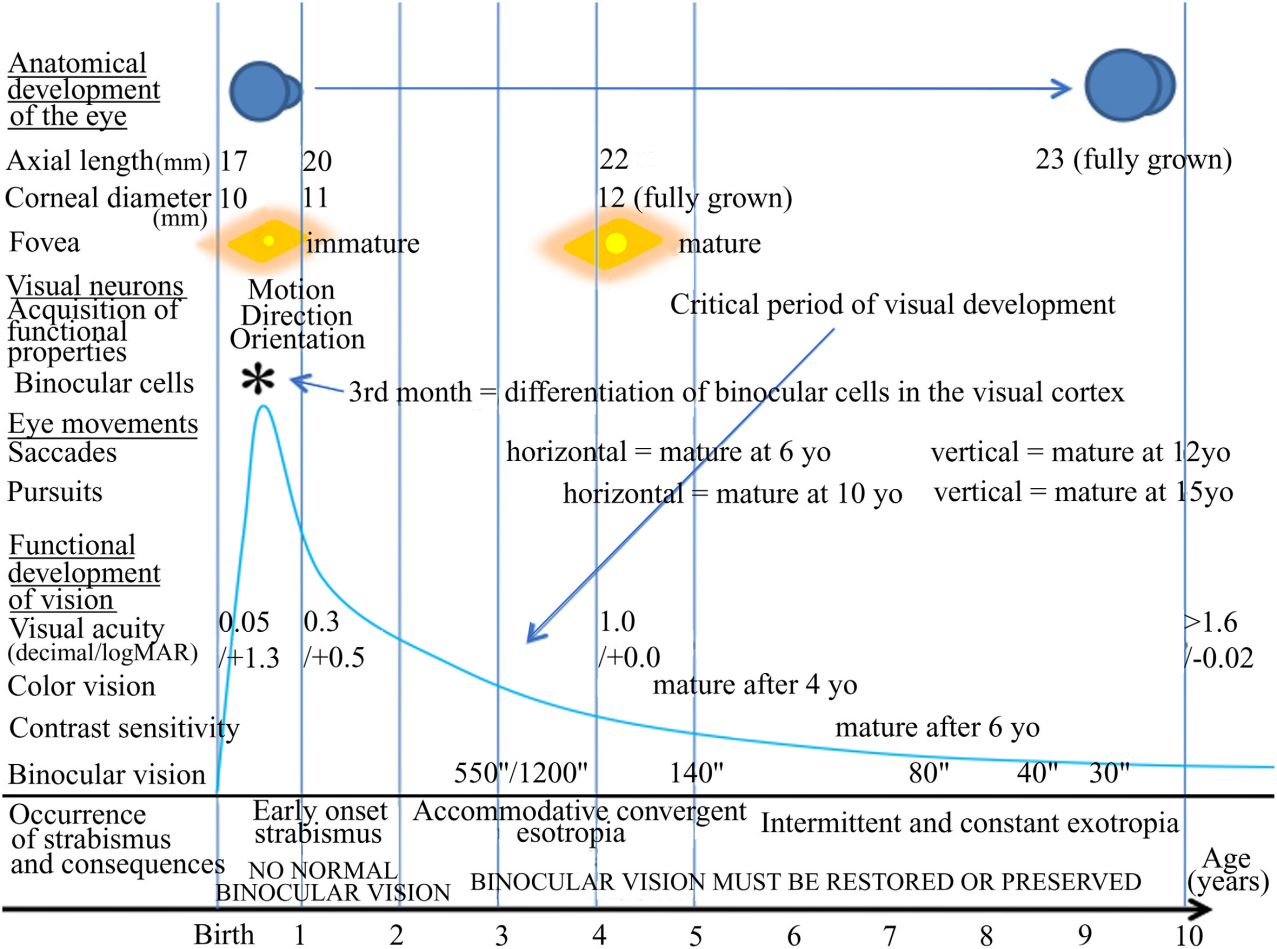
**Normal visual development in humans and times of occurrence of strabismus**. The normal development of vision in humans is characterized first by morphological changes including the growth of the eye, an increase of the corneal diameter and the formation of numerous connections between the eyes and the cortex that organize progressively with age. In parallel, functional changes occur. The retina matures, in particular at the level of the fovea. Neurons in sub-cortical and cortical structures also progressively acquire adult functional characteristics. Among such latter processes, neurons in primary visual cortex (V1) progressively acquire the capacity to be activated by one given orientation, one given direction of movement, one given velocity of the stimulus and to be activated through one eye and the other, thus becoming “binocular.” Eye movements such as saccades and pursuits also become “adult-like” with age but not at the same age. Altogether, this leads to the development of visual perception including acuity, color vision, contrast sensitivity, binocular vision and 3D perception. All of these processes occur during the so-called “critical period” of development, which corresponds to a period of high plasticity. This plasticity however changes with age, with its peak during the first postnatal year (as illustrated by the blue drawing). In case of abnormal vision, such as strabismus, during this period, the development of the visual system and of visual perception itself may be greatly altered, in particular regarding the development of an amblyopia and the loss of binocular vision. yo: years old. ^*^, 3rd month: differentiation of binocular cells in the visual cortex.

Visual perception is optimal in humans at adulthood, providing that all the developmental processes in relation to it have occurred properly both before and after birth, including anatomical and functional processes. As illustrated in Figure 1, this includes not only the correct development of the eyes themselves, but also that of eye movements through the extraocular muscles (EOMs). In parallel, all of the central structures in the brain that are related to visual perception (including those concerned with eye movements) must also develop appropriately. As a result, for example, each neuron in primary visual cortex (V1) becomes progressively able to encode the different attributes of the visual scene, such as orientation and direction of movement. Progressively, most of them also become “binocular,” i.e., able to be activated through both eyes while they are initially monocular (e.g., Frégnac and Imbert, 1978; Milleret et al., 1988). In parallel, cortical maps corresponding to each of these attributes of the visual scene, including the retinotopic map encoding space, develop (e.g., Chapman et al., 1996; Crair et al., 1998; Smith and Trachtenberg, 2007; White and Fitzpatrick, 2007; Tani et al., 2012 for review). The different types of eye movements (saccades, pursuits) also mature with age (e.g., Ingster-Moati et al., 2009; Bucci and Seassau, 2012, 2014; cf. Figure 1). The “quality” of both the postnatal visual experience and that of the eye movements play a major role in this, in particular during the so called “critical period” (e.g., Hubel and Wiesel, 1970; Buisseret, 1995 for review). At the end of all these processes, if they have occurred properly, an optimal visual perception in terms of acuity, color vision, perception of contrasts and binocular vision (which ensures 3D perception) is acquired progressively with age; cf. Figure 1.

Any alteration of postnatal visual experience and/or of eye movements during the critical period (which corresponds to the period of maximum plasticity) leads to the abnormal development of various structures in the brain, both anatomically and functionally. Strabismus is among these alterations. It has been identified for centuries and is characterized by a misalignment of the eyes. It presently affects approximately 2% of the human population worldwide. When occurring early in life, strabismus induces, for example, an abnormal development of both the geniculo-cortical pathway and interhemispheric connections through the corpus callosum (CC; e.g., Innocenti and Frost, 1979; Schmidt et al., 1997; Löwel et al., 1998; Bui Quoc et al., 2012). In parallel, neurons and neuronal maps in V1, as well as those in visual areas from the dorsal and the ventral streams, develop abnormal functional properties (e.g., Chino et al., 1983; Milleret and Houzel, 2001; Schmidt et al., 2004; Bui Quoc et al., 2012; see also Von Noorden, 1978; Milleret, 1994; Wong, 2012 for reviews). Importantly here, the binocular activation of visual cortical neurons (in V1 at least) is altered because of strabismus. Normally, these neurons receive progressively excitatory inputs from first the contralateral and then the ipsilateral eye during postnatal development (e.g., Frégnac and Imbert, 1978; Milleret et al., 1994). The neurons then potentiate each other to ensure binocular vision. Instead, after strabismus, neurons still sustain excitatory inputs from both eyes but the fixating eye neutralizes the neural response from the deviated eye through inhibition (e.g., Hubel and Wiesel, 1965: Singer et al., 1979; Chino et al., 1994; Sengpiel et al., 1994; Scholl et al., 2013). Binocularity is thus greatly disrupted. Although this phenomenon has been investigated less, neural bases for eye movements may also be abnormal, from the oculomotor muscles themselves to cortex. Altogether, this often leads to amblyopia and a loss of binocular vision (e.g., Sireteanu, 2000; Barrett et al., 2004; Birch, 2013 for reviews).

The question of the consequences of strabismus on both the neural bases of visual perception and visual perception itself has been widely investigated for decades and is still presently under investigation (e.g., Von Noorden, 1978; Milleret, 1994; Wong, 2012 for reviews). In contrast, the important question of the origins of strabismus remains poorly understood. In some cases at least, this may be extended to absence of binocular vision. Focalizing onto strabismus, it may display characteristics which vary greatly from one subject to the next. For example, it may be convergent or divergent. It may be horizontal or vertical. It may be intermittent or not. It may vary in amplitude. The age of its onset also may vary greatly (e.g., Donahue, 2007). We propose here that such “polymorphy” can only result from a multiplicity in the possible origins for strabismus. But, if so, what are these origins? Or, at least, what might they be? Our aim here is to attempt to advance the ideas surrounding that question. It is hoped that to answer such question will help in the improvement of the treatment of these pathologies in the future.

To our knowledge at least, the question of the origin of strabismus was first approached “scientifically” in the nineteenth century. Several theories were suggested. For example, Von Graefe (1854) insisted on mechanistic factors creating strabismus. Donders (1863) pointed out that refraction errors may have a role in the origin of strabismus through their links with accommodation. Duane (1869) proposed that it was an excess in vergence innervation that led to strabismus. Worth (1915) suggested that it was the absence of fusion of the images of both eyes that created strabismus, and that a “center of fusion” in the brain was implicated in this. Chavasse (1939) explained strabismus as a consequence of an excess in reflexogenic action. However, none of these mechanisms has even been proven. The same holds true regarding loss of binocular vision which may be either the consequence of strabismus or its cause. The absence of alignment itself prevents the development of normal binocular vision whereas, without binocular vision, the alignment of the eyes becomes unnecessary.

Nowadays, hypotheses on the etiology of strabismus have evolved and two major theories have thus emerged: a “sensory vs. motor” theory and a “peripheral vs. central” theory. The former theory proposes that strabismus may have a “sensory” or a “motor” origin, while the latter theory rather suggests that strabismus may have a “peripheral” or a “central” origin. The various forms of strabismus are therefore classified depending on those “sensory vs. motor” or “peripheral vs. central” oppositions.

The “Classification of Eye Movement Abnormalities and Strabismus” (CEMAS; http://www.nei.nih.gov/news/statements/cemas.pdf) is mainly based on the “sensory-motor” opposition. It directly and clearly differentiates strabismus from “other” eye movement abnormalities that would be not considered as “pure” strabismus. In the CEMAS, first, eye movement abnormalities or strabismus are defined as resulting from an abnormal motor system, thus having: (1) abnormal full versions and ductions, abnormal fusional vergence amplitudes; (2) non-accurate and abnormal speed saccades, abnormal gain pursuit and vestibular movements; (3) pathologic oscillations or intrusions. Second, a binocular sensory system is defined as abnormal if there is no bi-fixation with normal visual acuity in each eye, strabismus, diplopia, abnormal retinal correspondence, abnormal fusional vergence amplitudes, and abnormal stereopsis. It is also subnormal if there is one or more of the following characteristics: anomalous retinal correspondence, suppression, deficient to no stereopsis, amblyopia, and decreased fusional vergence amplitudes. Finally, the system is also considered as abnormal if there is no binocular vision. The complete classification of CEMAS is then described after those statements on motor and sensory aspects of the visual system. We shall not detail it here but only recall the following divisions: (1) horizontal heterotropias, either concomitant or non-concomitant, either divergent or convergent. In this section, the early onset esotropia, the nerve palsies, the accommodative esotropia and the constant or intermittent exotropias are also included; (2) horizontal heterophorias; (3) cyclovertical heterotropias and special forms of strabismus, i.e., oblique muscles palsy or dysfunction, restrictive strabismus and neuro-myogenic strabismus. In this latter case, myasthenia gravis, chronic progressive external ophthalmoplegia, internuclear ophthalmoplegia, and skew deviation are also classified. Special forms of strabismus are also mentioned in this section, such as co-contractive retraction syndromes, Restrictive Hypotropia in Adduction (RHA) and Congenital Fibrosis of the EOMs (CFEOM); (4) cyclovertical heterophorias; (5) accommodative disorders (Paralysis, Infacility, Insufficiency, Excess); (6) nystagmus and other ocular motor oscillations.

Contrasting with the former classification, the other way to classify rationally the different types of strabismus consists of segregating strabismus according to its “peripheral” vs. “central” origins. This is the classification which is presented in a recent synthesis achieved by Péchereau (2013). In his book, the author classified strabismus with a “peripheral” origin as those resulting from abnormalities at the level of the oculomotor muscles themselves or their innervation. These include, for example, the muscular dystrophies and the palsy of the 3rd, 4th, or 6th cranial nerves. Also included are the retraction syndromes (now called CCDD disorders for “Congenital Cranial Dysinnervation Disorders”), the Basedow disease (called “Graves disease” in the United States), and, finally, oculomotor abnormalities secondary to orbital fractures. In comparison, strabismus considered as having a “central” origin have also been classified. But the precise origins of those strabismus were not specified since that question has never been approached in the literature (at least from our knowledge). Among strabismus with a central origin, the author has included different types of strabismus depending on: (a) the type of deviation (vertical or horizontal strabismus, convergent or divergent, with or without eye cyclo-torsion); (b) the age of occurrence of strabismus (early onset until 6 or 8 months, late onset after 2.5 years, intermediate); (c) whether the deviation is constant or intermittent. He has additionally pointed out the importance of the analysis of binocular status when classifying strabismus, which depends on normal (or potentially normal) or abnormal binocularity. As indicated above, strabismus is most often associated with abnormal binocularity. This occurs in case of: (a) early onset constant divergent or convergent strabismus; (b) micro-strabismus caused by a hereditary absence of fusion; (c) evolution of a micro-strabismus in strabismus; (d) secondary strabismus (i.e., caused by an anatomical abnormality that decreases the vision of an eye). However, binocularity may be present in spite of strabismus in the following cases: (a) intermittent early onset strabismus; (b) late onset strabismus, convergent or divergent, intermittent or permanent; (c) accommodative strabismus, with or without excess or convergence; (d) latent strabismus (heterophorias). The author has also pointed out that oculomotor abnormalities may exist with or without deviation of the eyes (i.e., without strabismus), such as in the case of nystagmus without strabismus, or in the case of torticollis.

The “sensory vs. motor” and the “peripheral vs. central” theories in fact complement each other. This will be obvious from our further discussions below. Nevertheless, the precise “*primum movens*” of strabismus remains vague in both theories, and the very mechanisms and pathophysiology are rarely expressed. It is our main goal to open here the “black box” dealing with the origins of strabismus, in particular its central origins, and therefore we shall use the latter classification in the present article. In that regard, we shall tentatively take into account the most relevant present knowledge about the organization of the brain and its development. This knowledge will come from both biology and medicine. Some knowledge from anatomy, physiology, as well as genetics and molecular biology, will be thus considered. “Innate” and “acquired” factors which may potentially lead to strabismus and/or the absence of binocular vision will be also examined, in addition to peripheral vs. central factors and sensory vs. motor factors. Tychsen has stated already that it is the brain that must be repaired if ophthalmologists want to treat strabismus (Tychsen, 2005). We evidently agree with that idea but much still remains to be done prior to the complete treatment of strabismus. To treat consequences of strabismus on visual perception is already relatively effective, with conventional treatments including optical treatment with glasses, monocular occlusion, and alignment of the eyes through surgery. Newly developed strategies such as binocular training and transcranial magnetic stimulations (TMS) could improve in the future the efficacy of conventional strabismus treatment since it has been shown that such strategies permit the recovery of visual acuity and binocular vision in amblyopia, even at adulthood (after alignment of the eyes); (e.g., Nyffeler et al., 2006; Hess et al., 2010a,b; Hess and Thompson, 2013). Furthermore, considering strabismus at source and dealing in particular with its central origins is currently far from effective. The same applies to loss of binocular vision with a central origin. However, as a general rule in medicine, it is always better to treat pathology at source (provided its origin and pathophysiology are precisely defined) rather than dealing with its dilatory consequences. Our article aims at assisting in this regard by treating the question of the origins of strabismus, even if practical therapeutical consequences will not be immediate.

### Possible origins of strabismus: from the eyes to the brain

From the eyes to the brain, the visual system and the oculomotor system are both formed of complex neural networks which link numerous structures. Interactions exist between these structures within each system separately, as well as between both systems. Thus, all of these structures must function perfectly and in synchrony to ensure a normal visual function, i.e., the best possible acuity of each eye, a proper binocular (stereoscopic) vision, a normal alignment of the eyes and precisely shaped movements.

**Figure 2:**
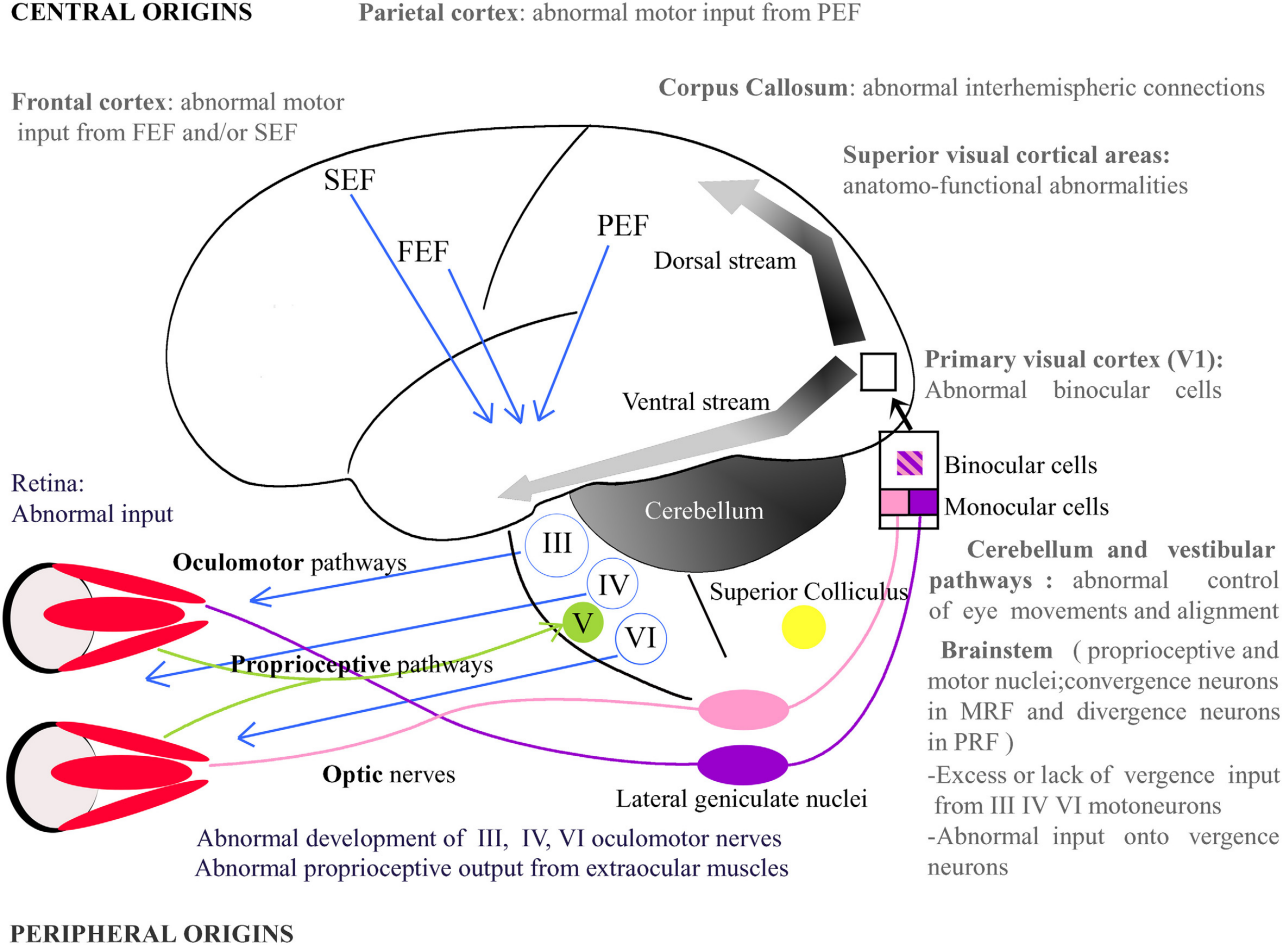
**Potential origins of strabismus**. Strabismus may have sensory and/or motor origins as well as peripheral and/or central origins. In periphery, one may notice, for example, abnormal vision or abnormal development of the extraocular muscles. In the latter, the extraocular muscles' proprioceptive afferents reaching the Gasser ganglion normally through the ophthalmic branch of the Vth (trigeminal) cranial nerve and/or their oculomotor nerves, i.e., the IIIrd, IVth, and the VIth cranial nerves, may be also altered. Centrally, strabismus may, for example, result from an abnormal activity in the brainstem, the Medial Reticular Formation (MRF), the Pontine Reticular Formation (PRF), the thalamus, the cerebellum or the superior colliculus. At the cortical level, visual, parietal or frontal cortex may also not function properly. Altogether, this indicates that origins of strabismus may be numerous. One may emphasize that those with a central origin likely dominate. FEF, Frontal Eye Field; SEF, Supplementary Eye Field; PEF, Parietal Eye Field; III, IV, V, VI: brainstem nuclei III, IV, V, and VI and their motoneurons.

In the visual system, as illustrated in Figure 2, the retino-geniculo-cortical pathway is the main sensory route that links the retina to V1: most ganglion cells of the retina project to the dorsal lateral geniculate nucleus (dLGN) via the optic nerves and the optic tracts. Geniculate cells then project to V1 through the optic radiations. From there, most of the afferents reach “superior” visual areas that form the “dorsal” and the “ventral” streams. Other afferents may interconnect both hemispheres through the CC. Any abnormality within one of these structures alters vision (e.g., Wong, 2012; Berlucchi, 2014 for reviews). Some fibers from the optic tracts also project to extra-geniculate structures which are themselves implicated in vision, such as the Superior Colliculus (SC). This structure, among other functions, is also responsible for a precise ballistic of the eye movements and for visual attention (Krauzlis et al., 2013).

The oculomotor system must also function perfectly to provide eye movements and alignment of the eyes with optimal characteristics. This begins with the six EOMs and their pulleys, which allow movements of the eyes: the external and internal rectus, the superior and inferior rectus, as well as the inferior and superior obliques. The motor activity of these muscles is controlled directly by the IIIrd (common oculomotor), IVth (trochlear), and the VIth (abducens) cranial nerves. During these eyes movements, some receptors in the EOMs (including their tendons) are also activated, sending sensory messages related directly to eye movements all around the brain, including within V1 and the frontal cortex (Buisseret and Maffei, 1977 for visual cortex; cf. Buisseret, 1995 for review) through the ophthalmic branch of the Vth (trigeminal) cranial nerve (e.g., Batini et al., 1975). Such proprioceptive information is relayed in different sub-cortical structures located in the brainstem, and also in the cerebellum and the vestibular nuclei, finally reaching the parietal and the frontal cortex (e.g., Fillenz, 1955; Batini and Buisseret, 1974; Donaldson and Dixon, 1980; Donaldson and Long, 1980; Ashton et al., 1988, 1989).

Our hypothesis here is that strabismus can be caused by an insult at every level of both the visual and the oculomotor systems. Below we shall thus describe different possible origins of strabismus at different levels of each system. Notice that many other possible origins might be proposed. The EOMs and their innervations (up to their first relay in the brain) will be considered at the “periphery,” while the eyes and the structures in the brain will be considered as “central.” Considering their embryonic origin, the eyes may indeed be considered as a prolongation of the brain.

## Peripheral and genetic origins of strabismus

As a first step, abnormalities at the level of the EOMs, whether genetic in origin or not, will be discussed as potential origins of strabismus. The potential role of the oculomotor muscles themselves in this process will also be discussed, as well as their sensory afferents or motor efferents.

### Abnormal weakness of EOMs

#### Muscular dystrophies, genetic myopathies, and myasthenia

Abnormal weakness of extraocular (or oculomotor) muscles may occur for various reasons. First, it may be due to muscular dystrophies or other genetic myopathies. In that case, the muscles themselves are affected, which can cause strabismus (Shieh, 2013). The most common forms of muscular dystrophies include Duchenne muscular dystrophy, Becker muscular dystrophy, facio-scapulo-humeral muscular dystrophy and Steinert myotonic dystrophy. Variable phenotypes of strabismus and abnormalities of ocular movements are often associated with such muscular dystrophies. Second, it may be the transmission of neurotransmitters at the level of the neuromuscular junction that is affected in myasthenia which may lead to variable and intermittent forms of strabismus. Moreover, EOMs, being the most fatigue-resistant muscles of the body, can therefore be less or lately affected by a muscular dysfunction.

#### Strabismus and pulleys

The understanding of eye movements have been deeply modified by Demer's work, which described the role of the pulleys in the kinematics of EOMs (e.g., Demer, 2003; Demer et al., 2008). Abnormal position or function of those pulleys may lead to strabismus.

### Abnormal development of the innervation of EOMs in various forms of complex incomitant strabismus

#### Abnormal motor innervation of the EOMs

Congenital fibrosis of the EOMs (CFEOM) leads to a first form of strabismus due to abnormal motor innervation. This affects patients with congenital restrictive ophthalmoplegia (Engle, 2006; Graeber et al., 2013). CFEOM is a misnomer for various incomitant strabismus which were described in several families, indicating a possible hereditary trait. Indeed, genetics have proven that CFEOM result from mutations in genes that are implicated in the growth of oculomotor nerves. In the absence of normal innervation, a variable atrophy of the muscles may occur. Several forms of CFEOM exist. CFEOM 1 results from KIF21A mutations and is characterized by a quasi total absence of movement of the eyes, the primary position being hypotropic with elevation being impossible, whereas the horizontal position can be either esotropic or exotropic. The heterozygous mutation of the gene is sufficient to cause the disease which results in a dominant inheritance. KIF21A is located on chromosome 12 and is responsible for the production of a developmental kinesin. Kinesins are molecular motors that interact with and transport cargo along the microtubules of axons. In CFEOM 1, abnormal development of the superior branch of the IIIrd nerve particularly affects the function of the superior rectus muscle (and the levator palpebrae superioris muscle). In CFEOM 2, the phenotype results from an insult to all branches of the IIIrd and IVth nerves. Patients with CFEOM 2 have their eye in the exotropia position with no movement possible. It is mutations in gene PHOX2A, located in 11q13 that cause the disease. PHOX2A is a paired-linked transcription factor gene and its expression is restricted to differentiating neurons in the central and peripheral nervous systems. Therefore, the pathogenesis of CFEOM 2 would be an abnormal development of both the IVth and the IIIrd nerves. In CFEOM 3, similar to CFEOM 1, it is only the development of the IIIrd nerve that is affected, although in a more severe way since all of its branches are affected in this situation and not only those branches which innervate the superior rectus muscle and the levator palpebrae superioris muscle. The gene responsible for the disease is located at 16qter.

Contrarily to CFEOM syndromes, in which development of the III^rd^ nerve is affected, it is the development of the VIth nerve which is affected in the Duane syndromes. The *primum movens* of these latter syndromes is an absence of development of the VIth nerve, either unilaterally or bilaterally. Variable forms of abnormal innervation of the lateral rectus muscle by branches of the IIIrd nerve have also been observed, resulting either in esotropia or exotropia. Again, it is an abnormal development of a nerve that underlies the pathogenesis of this condition, with genes located at 8q13 and at 2q31. Duane Radial Ray Syndrome (DRRS) is a particular form of Duane Syndrome in which the ocular movement abnormality is associated with bone abnormalities in the hand, such as an absence or a malformation of the thumb which can look like a finger. Again, it is a mutation in a transcription factor gene SALL4, located at 20q13, which causes the disease by altering the normal neural development.

Other insults to the development of the VIth nerve include HGPPS (Horizontal Gaze Palsy with Progressive Scoliosis), in which it is mutations of the gene ROBO3, located at 11q23, which are responsible for the resulting phenotype. Such phenotype combines a total impossibility of horizontal gaze movements, along with a scoliosis that occurs during the first decade of life. ROBO3 is a developmental gene and is expressed in the hindbrain of the human fetus. Human ROBO3 is similar to roundabout genes that are responsible for axon guidance in other species such as mice, zebrafish or drosophile. Indeed, brainstem neurons of ROBO3^−/−^ mice completely fail in crossing the midline during development (Marillat et al., 2004). In zebrafish, and in drosophile, the loss of function of ROBO3 results in aberrant midline crossing by axons (Seeger et al., 1993).

Finally, abnormal development of the VIth nerve occurs in two conditions in which mutations of the gene HOXA1 (located at 7p15 and implicated in hindbrain segmentation during fetal development) are responsible: the Bosley-Salih-Alorainy syndrome (BSAS) and the Athabascan Brainstem Dysgenesis syndrome (ABDS). In BSAS, which is a recessive condition, a bilateral Duane trait is associated with other cranial nerve dysfunctions, such as deafness due to a bilateral absence of the cochlea and misdevelopment of the VIIIth (vestibulo-cochlear) nerve. In ABDS, horizontal gaze restriction is associated with central deafness and mental retardation.

The various abnormalities of the development of the IIIrd, IVth, or VIth cranial nerves have been regrouped in a generic term: CCDD (see above). In CCDDs, the insult can cause ocular movement disorders but other conditions without strabismus can occur, such as isolated congenital ptosis which can result from a mutation in a gene located at 1p34-p32. As a general rule, genetics of CCDDs teach us that an abnormal development in general and an abnormal early routing of neurons in particular, may cause strabismus. In CCDDs, the insult occurs at the frontier between peripheral and central locations. It is the same when considering the cranial nerves that emerge from the brainstem and lead toward abnormal EOMs. The model of the CCDDs therefore emphasizes that an abnormal neural network can cause strabismus.

Palsy of the IIIrd, IVth, or VIth nerve leads to a second form of strabismus. Similar to CCDDs, it is a neural disorder although the cause is often acquired and not innate. The nerve palsy causes an atrophy of the innervated muscle. Acute palsy of the IIIrd nerve is an emergency since it can be caused by a direct compression of the nerve by a cavernous sinus thrombophlebitis, or by an aneurysm of the posterior communicant artery. A palsy of the IVth nerve is either congenital or acquired. When acquired, it is a peripheral cause that is the *primum movens*. It may however result from a direct insult after a severe cranial traumatism. A palsy of the VIth nerve may finally result from a direct compression of the nerve's fine and fragile branches. This can arise from a hypertrophic brainstem due to intracranial hypertension. It may also result from numerous other central causes such as tumors, infections, etc. In all cases however, this will induce strabismus.

#### Strabismus and extraocular proprioception

The outflow theory supports the idea that it is an efferent copy of the oculomotor signal from the motor centers that gives information about the position of the eyes to the brain (Von Helmholtz, 1866). By contrast, the inflow theory claims that it is the proprioceptive signals from eye muscle receptors that give such information (Sherrington, 1918). More recent experiments support one theory or the other. Thus, authors now consider that both theories are right and that efferent copy co-exists with extraocular proprioception. Proprioceptive receptors do exist in the EOMs, in particular at the level of the tendons (e.g., Cooper and Daniel, 1949; Richmond et al., 1984 for receptors in humans). These receptors are active and send sensory messages to numerous regions in the brain implicated both in visual perception and eye movements (e.g., Donaldson, 1979; Donaldson and Dixon, 1980; Milleret et al., 1987). Furthermore, they have been demonstrated by our group to strongly contribute to the maturation of visual neurons in V1 during development (cf. Buisseret, 1995 for review; see also Buisseret et al., 1978, 1988). Thus, an abnormal proprioception at the level of the EOMs is also a potential cause for strabismus, since abnormal information about the position of the eyes leads to an abnormal central and neural motor command in return.

Nevertheless, a deafferentation of EOMs has never been demonstrated to affect ocular motor control and to induce strabismus. To our knowledge at least, neither experimental research nor any medical cases have demonstrated this (the problem is a difficult one to approach). Thus, some authors have concluded that proprioceptive signals only play a role during development in calibrating the efferent copy signal, which is sufficient to provide information about eye movements and position (Lewis et al., 2001). Other authors, however, have claimed that an insult to proprioceptive receptors of the EOMs could be the cause of strabismus (e.g., Donaldson, 2000). Similarly to the CCDD (see above), abnormal development of the proprioceptive axons within the Vth cranial nerve to the Gasser ganglion may occur. It could also be hypothesized that abnormalities of the extraocular receptors could be responsible for strabismus. This is supported by investigations of some strabismic patients whose extraocular muscle receptors display abnormal morphological characteristics (Li and Shen, 2001). These changes were analyzed using transmission electron microscopy and revealed both a decrease in the number of mitochondria in axons, and the disappearance of the nerve component of the receptor. Of course, in such a study, whether the abnormalities in the proprioceptors are the cause of strabismus or its consequences cannot be distinguished. This recalls the controversy regarding whether the subtle changes at the cellular level of the muscles (especially the singly innervated orbital fibers) of strabismic patients can be the *primum movens* of strabismus, or are simply an adaptative phenomenon to the deviation (Lennerstrand, 2007). Finally, the implication of extraocular proprioception during ocular movement disorders can be emphasized by the fact that a tenotomy of all of the EOMs and their reattachment, which suppresses the proprioceptive output signals, is an effective therapy in the treatment of some forms of infantile nystagmus (Dell'Osso and Wang, 2008).

### Genetics of concomitant strabismus

Contrary to CCDDs or to nerve palsies, most strabismus such as congenital strabismus, accommodative strabismus or divergent strabismus are concomitant, meaning that the deviation is always the same whatever the gaze direction; they also likely display a central origin (see below). Also contrary to CCDDs, no single gene has been identified as the direct origin of concomitant strabismus. Nevertheless, inheritance and genetics are obvious in the development of most forms of strabismus, either incomitant or concomitant (Engle, 2007). However, additional factors, in particular those related to developmental processes, also need to be taken into account.

Hippocrates himself would have stated that: “squinters engender squinters.” Physicians are evidently also aware of the influence of genetics on strabismus and usually advise strabismic parents that their children must be screened for strabismus. Indeed approximately 15% of children of strabismic parents are strabismic, compared to the 2% prevalence of strabismus in the general population (Donnelly, 2012). Ziakas showed however that this proportion may vary depending on the type of strabismus and the degree of relationship (Ziakas et al., 2002). In his study of 96 index cases with strabismus with either early onset strabismus (26 cases), accommodative esotropia (49 cases), anisometropic esotropia (15 cases), or exotropia (56 cases), he showed that the risk of having strabismus for a first degree relative is 4% for exotropia but 26.1% for accommodative esotropia. In accommodative esotropia, the risk decreases to 7.5% for second degree relatives and to 4.8% for third degree relatives. In twin studies, it has been shown that there is a specific genetic influence for eso-deviation which is independent of the refractive error (Sanfilippo et al., 2012). The heritability of eso-deviation is estimated as 64% in a cohort of 1462 twin pairs with a prevalence of 8.6% of eso-deviation, the correlation being significantly greater in monozygotic twins (*r* = 0.65) than in dizygotic twins (*r* = 0.33). But, as indicated above, the genetic contribution to concomitant strabismus is not easy to circumscribe. Thus, even in the case of “simple” strabismus such as early onset esotropia, accommodative esotropia or exotropia, genetic inheritance is complex, with the possible implication of recessive genes as well as dominant genes.

The influence of the degree of development of both the eyes and the brain at birth must also be taken into account. Recalling the higher proportion of strabismus in premature infants compared to full term infants illustrates this (Torp-Pedersen et al., 2010). This emphasizes the relationship between the development of the brain (including the eyes) and the potential development of strabismus after birth. More generally, this indicates that an abnormal (or an immature) development of the neural networks, resulting from innate (i.e., genetic) or acquired factors, might, in turn, lead to strabismus.

## Possible central origins of strabismus

As indicated above, it is the anatomo-functional maturation before and after birth of multiple neural networks from the eyes to the brain that subtend the normal development of visual perception. This occurs by implicating both genetic and epigenetic factors such as postnatal visual experience. Our driving hypothesis here is that any insult to this normal process of maturation may, in turn, generate strabismus. This applies evidently to any level of the sensory and/or motor networks that are involved in the elaboration of visual perception (cf. Figure 2). Some examples are provided below to illustrate this. How an abnormal development of any visual path or any neural activity somewhere within the visual system may lead to strabismus are considered in succession. How an abnormal neural activity in the oculomotor system may lead to strabismus is also discussed.

### Abnormal development of the visual paths

First, we hypothesize that any insult during the normal processes of neurogenesis, axonal growth, migration of neurons, synaptogenesis, myelination, apoptosis or even elimination of juvenile exuberant axons, may potentially lead to strabismus. For example, strabismus may be the consequence of the misrouting of some paths within the visual and/or the oculomotor networks.

#### Abnormal routing of ganglion cell axons

Interestingly, Siamese cats spontaneously display a convergent strabismus. They also have an abnormal predominance of the crossed retino-geniculo-cortical pathway compared to normal cats (Montero and Guillery, 1978; Shatz and Levay, 1979). This results from stagnation at an early stage of development, which itself recalls the development of visual pathways during phylogenesis. We propose that such abnormal predominance of the crossed retino-geniculo-cortical pathway may also be the cause of the early onset convergent strabismus in humans, in which the early asymmetry of the optokinetic nystagmus also persists with age.

Paradoxically, in case of divergent strabismus, it could also be hypothesized that a predominance of the crossed pathways could be the *primum movens* of strabismus. During evolution, the visual system is first an “only crossed fibers” network with lateral eyes and panoramic vision. It then evolves to a balanced system with equal importance between the direct pathway and the crossed pathway, and frontal eyes allowing binocular vision. We propose here that an abnormal routing of the retinal ganglion cell axons at the level of the optic chiasm might lead to a loss of balance between crossed and direct fibers and thus lead to strabismus. This might occur by an abnormal expression of ephrins, i.e., surface molecules which are specifically implicated in guiding the retinal ganglion cell axons at the level of the optic chiasm during the developmental process (Petros et al., 2009). The axons of ipsilateral projections from temporal retina (direct fibers) express the guidance receptor ephrin B1 (but not the axons of contralateral projections from the nasal retina, i.e., crossed fibers). At the optic chiasm, radial glia cells express ephrin B2, which repulses the ephrin B1 axons from crossing the midline, unlike the contralateral fibers from the nasal retina. Expressions of ephrins and of ephrin receptors are specific and precise timing is necessary to ensure the normal and balanced development of visual pathways. If this system was altered through an abnormal expression of ephrins and/or ephrin receptors during development, an asymmetrical neural network of crossed and uncrossed fibers would develop and could result in the development of strabismus.

#### Misrouting and abnormal retinotopy

During development, ganglion cell axons reach progressively central visual structures by respecting “retinotopy.” The visual field is thus encoded by neurons with precision from the retina up to the cortex (e.g., Tootell et al., 1998 for review), a necessary condition to ensure normal visual perception, including binocular visual perception. Guidance of axons creating retinotopy is also permitted by ephrins. Gradients of ephrins A and ephrins B, both in the retina and in the visual cortex, allow the creation of x and y coordinates (Cang et al., 2005a,b). This leads to the establishment of neuronal “retinotopic maps,” which are refined with age and visual experience. Again we propose here that abnormal guidance through abnormal levels of ephrins A or B and/or their receptors during development would alter retinotopy and would cause, in turn, strabismus. In some cases at least, distortions within retinotopic maps, which may lead to abnormal retinal correspondence in early onset strabismus (e.g., Wong, 2000; Popple and Levi, 2005; Mansouri et al., 2009; Wang et al., 2012), would therefore be a cause of strabismus rather than a consequence.

The subplate, i.e., mostly temporary cells located below layer VI of Area V1, plays a major role for growing axons to reach the visual cortical plate during development (Ghosh et al., 1980; McConnell et al., 1989). Any abnormality during such process would interfere with normal development of geniculo-cortical connections, thus with normal development of retinotopic maps in V1. Again, this could potentially induce strabismus.

#### Abnormal cortico-cortical connections

Strabismus is now well known to disrupt the development of numerous cortico-cortical connections implicated in visual perception. These cortico-cortical connections may be “short” and located within one given area or “long,” thus linking various areas which may be located very far from one to the other. Thus, for example, strabismus is known to stabilize normally transient intra-hemispheric cortico-cortical connections in V1, leading to interconnect larger cell groups driven through the same eye than in the normal case (e.g., Löwel and Singer, 1992; Schmidt and Löwel, 2008). It is also known to lead to drastic anatomo-functional changes in the organization of the interhemispheric callosal connections, which normally link reciprocally and homotopically various visual areas to “glue” both visual hemifields into a single scene (Payne, 1990, 1991; Payne and Siwek, 1991a,b; Bui Quoc et al., 2012). In particular, in the case of strabismus, it leads to the development of asymmetrical interhemispheric connections which prevent the fusion of both visual hemifields along the vertical midline (Lund and Mitchell, 1979; Milleret and Houzel, 2001; Bui Quoc et al., 2012).

Our idea here is that abnormal anatomical cortico-cortical connections within or between visual cortical areas (whatever their origin) may conversely lead in turn to strabismus. Our hypothesis may be supported first by experiments which have consisted in cutting the CC of adult cats, who rapidly displayed a misalignment of their eyes and even strabismus (Elberger, 1979; Payne et al., 1981; Elberger and Hirsch, 1982). This is further supported by studies showing the implication of the CC during eye movements (e.g., Pasik et al., 1971; Tusa and Ungerleider, 1988; Zernicki et al., 1997). Our hypothesis is also strengthened by analyzing the deficits in visual and visuo-spatial developments that are present in young children with Williams syndrome. They are interpreted as the result of a split between the ventral and dorsal stream processing of visual information (see Figure 2), with a generalized deficit in dorsal stream processing (Atkinson et al., 2001). Of great interest here, the authors underlined that patients with such syndrome also display a much higher incidence of strabismus, visual acuity loss, amblyopia and reduced stereopsis than the general population.

### Abnormal development of neuronal activity

In addition to genes, axonal guidance cues and molecules, spontaneous and early visually evoked neural activity are necessary for anatomical and functional refinement of developing visual circuits (e.g., Huberman et al., 2008 for review). Appropriate synchronizations within the visual network then need to develop in order to elaborate visual perception optimally (e.g., Singer, 1999, 2013; Uhlhaas et al., 2009a,b; Menon, 2013). Any abnormality in such neural activities from the retina to the visual cortex may also lead to strabismus. Some data in the literature strengthens this idea already. The same applies to neural circuits subtending eye movements.

#### Effects of an abnormal neuronal activity on visual system

Let us evaluate, in succession, the potential impacts on the alignment of the eyes of: (a) abnormal prenatal retinal waves; (b) abnormalities during postnatal visual experience; (c) abnormal excitation/inhibition balance; and (d) pathological asynchrony of neural activity.

- **Abnormal retinal waves**. First, prenatal spontaneous neural activity in retina, discovered by Galli and Maffei (1988), must be absolutely normal to allow the visual system to organize with precision. It plays both permissive and instructive roles. Indeed, even if this activity is generated very early in life, before vision begins, it is a necessity for the proper development of functional properties of visual neurons and that of the various functional maps all along the visual system. The retinotopic organization of the retino-geniculo-cortical projections is affected first. Indeed, retinotopic maps in the SC, dLGN, and V1 all develop before photoreceptors can be driven by light. The same applies to eye-specific inputs to dLGN and ocular dominance columns in V1. Orientation-selective circuits in V1 also start to form before visual experience begins. The same applies to circuits encoding spatial frequency (Tani et al., 2012). This is possible because spontaneous neural activity in the retina is highly structured, and thus allows the transmission of very precise messages to the central nervous system. This is achieved through slow wave oscillations with very specific spatial and temporal characteristics (Rochefort et al., 2009). If retinal waves display abnormality, for any reason, this entire process of development will be disrupted. The segregation of inputs from both eyes and/or the development of retinotopic maps will be abnormal (e.g., Cang et al., 2005a,b; Xu et al., 2011; Ackman et al., 2012; see also Huberman et al., 2008 for review; Figure 1). The orientation and/or the spatial frequency maps might also develop incorrectly. In other words, prenatal neural bases for binocular integration and/or for acuity would be altered centrally. It must also be taken into account that such alterations may in turn lead to misalignment of the eyes. For example, this may occur through incongruent interactions with the oculomotor system. As discussed below, this may also occur during development of visual perception itself. Even if it is difficult to prove, such a possibility might unavoidably correspond to the etiology of some forms of strabismus at least.
- **Abnormal visual perception may induce strabismus**. Coming back to the normal process, once the visual system becomes capable of responding to light, sensory-evoked activity then stabilizes the nascent visual connections, refines them further or induces additional circuit properties (e.g., Huberman et al., 2008 for review). But any abnormality within the visual network (because of abnormal retinal waves prenatally or otherwise) will again lead to an abnormal visual perception, with a central origin. Thus, for example, an abnormal segregation of inputs from both eyes and/or an abnormal retinotopic map will lead to an abnormal binocular integration. Abnormalities in the orientation and/or the spatial frequency maps will lead to amblyopia, which may itself lead to strabismus, because of the poor ability to fixate of the amblyopic eye during binocular fixation. A decrease in vision is very well known to impair the proper alignment of the eyes (e.g., Quick et al., 1989). Amblyopia can also be responsible for abnormal saccades and pursuits (e.g., Niechwiej-Szwedo et al., 2010). Considering more extreme conditions, blind people also systematically display completely uncorrelated eye movements.
- **Abnormal balance excitation/inhibition**. The visual system is a complex network of neurons interconnected through excitatory or inhibitory synapses. Suppression is as important as activation, in particular postnatally. Thus, for example and of great importance here, it has been demonstrated that interocular suppression occurring in V1 of strabismic patients involves GABAergic-mediated inhibition (Sengpiel et al., 2006; see also Scholl et al., 2013). It has also been shown recently that it is the transformation of parvalbumin GABAergic (PV) interneurons from excitatory neurons to inhibitory ones that opens the critical period of visual development by internalizing the homeoprotein Otx2 (Sugiyama et al., 2008; Beurdeley et al., 2012). Reducing intraocular inhibition in the adult visual cortex has also been demonstrated to promote plasticity (e.g., Harauzov et al., 2010). In short, a balance between excitatory and inhibitory inputs from retina to cortex is required for elaborating correctly visual perception. Abnormality in this balance, either before or after birth, might lead to abnormal vision and/or uncorrelated eye movements.
- **Abnormal synchronization of neural activity**. The oscillatory pattern of neuronal responses and the synchronization of the oscillations from retina to cortex are now considered as playing a major role in elaborating visual perception (e.g., Gray et al., 1989; Engel et al., 1991; Neuenschwander and Singer, 1996; Castelo-Branco et al., 1998; Fries et al., 2002; see also Singer and Gray, 1995; Singer, 1999, 2013; Engel et al., 2001 for reviews). We propose here that any abnormality of this synchronization, at any level, may lead to strabismus (as well as binocular vision loss and/or amblyopia).
  Visual cortex is a highly distributed system implicating more than forty areas distributed from the occipital lobe to the parietal and temporal lobes (Figure 2). To elaborate visual perception, these areas operate in parallel and interact with one another to complement each other. This is achieved through short and long cortico-cortical connections which allow synchronizing of oscillatory neuronal responses within each area and between different areas, mainly in the β and γ frequency range i.e., 20–100 Hz (Engel et al., 2001; Fries, 2005). Among other functions, this is considered as solving the “binding” problem which consists in assembling all the attributes of the visual scene (namely location in space, direction of movement, orientation, spatial frequency, disparity etc.) into a coherent form during visual perception in various contexts, attention states etc. (e.g., Singer, 1999, 2013; Fries, 2005 for reviews). Very recently, this has also been established to allow prediction of perception (Hipp et al., 2011).
  Such synchronization develops with age, at least up to adolescence, in parallel to the maturation of cortico-cortical connections (including their myelinization) as well as excitatory and inhibitory circuits (Uhlhaas et al., 2009a,b). The maturation of the inhibitory PV neurons again plays a major role in this process since they serve as “pacemakers” for rhythmic neuronal activity, in particular in the γ frequency range (30–100 Hz). In other words, they assume a pivotal role in the temporal structuring and coordination of neuronal responses (Cardin et al., 2009; Sohal et al., 2009). Of interest, without going into details, all this developmental process of the brain rhythms occurs under strong genetic control (e.g., Buzsáki et al., 2013 for review). Simultaneously, visual perception also increases. Thus, for example, Csibra et al. (2000) measured γ band responses in EEG data in 6- and 8-month old infants during the perception of Kanisza squares that require the binding of contour elements into a coherent object representation. Based on prior behavioral studies that showed that infants up to 6 months of age are unable to perceive Kanisza figures, the authors hypothesized that perceptual binding in 8-month-old infants is related to the emergence of the γ band oscillations.
  Not surprisingly, epigenetic factors also play a role in this. Thus, any abnormal postnatal visual experience such as the one resulting from strabismus modifies the normal development of synchronization within the visual system by altering both wiring and neural activity (e.g., Löwel and Singer, 1992; Schmidt and Löwel, 2008). Neuronal synchrony is reduced in visual cortex compared to normal (Roelfsema et al., 1994). Recent data from Hess and his group have strengthened this by establishing that interactions between cells in disparate brain regions are reduced when driven by the amblyopic eye of strabismic subjects, from dLGN to superior visual areas, via V1 (Li et al., 2011). They have also demonstrated that amblyopia (in strabismic patients) is associated to temporal synchrony deficits (Huang et al., 2012).
  In turn, we postulate that any abnormality within one given visual area or between at least two visual areas, due to developmental anatomical and/or functional abnormalities somewhere in the visual system, may lead to strabismus (and amblyopia and/or binocular vision loss) by altering synchrony. Since abnormalities in synchrony may occur before or after birth (see above), this may lead to an early or a late strabismus. The same idea may be extended to the oculomotor system since it has been shown recently that it has its own dynamics (Gregoriou et al., 2012; Cordones et al., 2013) and that changes in neural synchrony also occur during development of the motor system (Kilner et al., 2000). Situations when the visual and the oculomotor systems need to interact to elaborate visual perception, i.e., during sensori-motor processing, are also affected. One may underline that our hypothesis is directly in line with increasing evidence that disturbances of synchrony in the developing brain, associated to aberrant neurodevelopment, subtend the cognitive dysfunctions associated to major brain pathologies such as schizophrenia and autism spectrum disorders (Uhlhaas and Singer, 2006; Uhlhaas et al., 2009a,b, 2011). As outlined above, genetics play a major role in that. Thus, for example, in schizophrenic patients, the GABA synthesizing enzyme GAD 65 and the calcium-binding protein parvalbumin are down-regulated in basket cells, while they are crucial for the generation of γ rhythms (Lewis et al., 2005; see also above).

#### Effects of abnormal neural activity on oculomotor system

Neuronal activity within the various structures implicated in the movement of the eyes also needs to be normal whatever the age. As illustrated below, any abnormality may induce strabismus.

- **Abnormal extraocular proprioceptive afferents from EOMs to V1**. Proprioceptive afferents from EOMs project to V1 (Buisseret and Maffei, 1977). They strongly contribute to the maturation of visual neurons in V1 during development, including their ability to perceive details. Thus, when removed in their entirety early in life, visual neurons do not develop their functional properties properly. It is as if they had never benefited from any visual experience. Also, if proprioceptive afferents in one plane are removed, a perpendicular meridian amblyopia develops (cf. Buisseret, 1995 for review). Since an amblyopia may induce strabismus (cf. above), we put forward the idea that any disequilibrium in the proprioceptive afferents from the EOMs might also induce strabismus. Note that such a process might be extended to any of the central structures which receive afferents from the EOMs, belonging to both the visual and the oculomotor systems (e.g., Donaldson, 1979; Donaldson and Dixon, 1980; Milleret et al., 1987).
- **Abnormal activity of the vergence neurons and abnormal cortical control**. Specific convergence neurons have been identified in the Medial Reticular Formation of the brainstem (Mays, 1984) and abnormal activity, either an excess of activity or a loss of activity, may also be responsible for a deviation of the eyes. Hyperactivity of these neurons could lead to convergent esotropia. Hyperexcitability of the neurons, enhancing the accommodation/convergence loop, may play a role in accommodative esotropia with an excess of convergence. On the other hand, a loss of activity of the neurons, premature apoptosis or a progressive degeneration of the neurons or axons may also induce exophoria and exotropia. Such a progressive insult to the system would explain the natural history of divergent strabismus, in which there is an increase in divergent deviation with time.
  Similarly, divergent strabismus may result from an excess of positive inputs from the divergence neurons which have been identified in the Pontine Reticular Formation (cf. also Mays, 1984). It has been hypothesized that a lack of activity of those divergence neurons would induce esotropia.
  Higher structures command eye movement and eye position. Our hypothesis here is that the genesis of strabismus may also result from abnormal inputs from those cortical structures which play a role in the triggering of ocular movements such as Frontal Eye Field, Supplementary Eye Field, and Parietal Eye Field (cf. Figure 2). Indeed, it has been shown by neuro-imaging that, in adult strabismic patients, the gray matter volume of those cortical eye fields can be abnormal, either larger or smaller (Chan et al., 2004).
- **Abnormal activity in Superior Colliculus, cerebellum and vestibular pathways**. The SC is a key structure in the control of eye movements. It is another structure that may potentially contribute to inducing strabismus. For example, it has been hypothesized that an insufficiently developed neuronal coupling between both superior colliculi would be implicated in vertical dissociated deviation, which is a particular form of strabismus that is associated with early onset strabismus (Brodsky, 2011; Ten Tusscher, 2011). Cerebellum and vestibular nuclei also control eye movements in normal visual conditions. An insult to those structures could also be hypothesized to be a central cause of strabismus. This is supported by the fact that an insult to the vestibulo-ocular input, through an attempt at the level of the otoliths, can cause this particular vertical strabismus, known as a “skew deviation” (Schlenker et al., 2009). Also, a malformation of the cerebellum, such as the one found in Joubert syndrome or in rhombencephalosynapsis, is associated with strabismus (Canturk et al., 2008; Keskinbora, 2008).

## Conclusion

For the first time, at least in our knowledge, instead of treating the question of the consequences of strabismus in humans, our article highlights the question of the *origins* of strabismus. The “polymorphy” of strabismus indeed suggests multiple origins but most of them are presently unknown. Those strabismus with a peripheral origin are rather well characterized but they are only few. In contrast, the other forms of strabismus, which are considered as having a *central* origin, are poorly understood despite being the most frequent. At present it is as if these latter forms of strabismus are included in a “black box” that has never really been opened by anyone. To move forward, we have decided here to tentatively open this box. We have proposed mechanisms which show that a central abnormality may lead to the development of strabismus. Our hypothesis on the mechanisms of strabismus are based on both classical and the most recent knowledge about the development and the organization of the visual system in mammals both before and after birth. Research in that field has indeed been very active all around the world for decades and is still very active today, providing numerous “keys” which might open our black box. Some other mechanisms are based on knowledge regarding the oculo-motor system from EOMs to cortex. In that context, we have also revisited the question of the origins of binocular vision loss, with tentative new ideas on the question. Interestingly, whether the origins of strabismus or those of binocular vision loss are considered, some of the new mechanisms we propose are already supported by published data. Altogether, our findings clearly emphasize the necessity to develop and to apply as soon as possible new strategies to treat strabismus and binocular vision loss, in particular through “central” therapies, in addition to the peripheral ones.

### About the origins of strabismus

Origins of strabismus with a peripheral origin are rather well known but they represent less than 5% of strabismic patients. As discussed above, they display either rare forms of incomitant or concomitant strabismus. As illustrated in Table 1, it is possible to distinguish which origins may induce early strabismus (up to 8 PN months) and/or late strabismus (from 24 PN months). Thus, an abnormal weakness of EOMs, such as the ones due to muscular dystrophies, myopathies, myasthenia or abnormal muscular pulleys, may induce both forms of strabismus. In contrast, abnormal development of the innervation of the EOMs, either motor or proprioceptive, may only induce an early strabismus. Not surprisingly, innate nerve palsies and acquired ones may induce early and late strabismus respectively. Of interest, it is also already established that most forms of strabismus with a peripheral origin have a genetic origin. As summarized in Table 2, most genes associated to such forms of strabismus have been successfully identified. This has been facilitated by the fact that the alteration of only a few specific genes is generally associated to each specific disease leading to strabismus. Thus, for example, Duchenne muscular dystrophy leading to the weakness of EOMs implicates only the DMD gene located on Xp21.2. The abnormal innervation of the EOMs in the context of HGPPS syndrome only implicates the ROBO3 gene located on 11q23.

**Table 1:** **Peripheral and likely central origins of strabismus with early or late onset**.

**For early onset of strabismus (at less than 8 PN months)**		**For late onset strabismus (at more than 24 PN months)**
**PERIPHERAL ORIGINS:**	Abnormal weakness of EOMs:	
Rare forms of incomitant or concomitant strabismus (<5%)	– Muscular dystrophies, genetic myopathies and myasthenia – Abnormal muscular pulleys	
**Abnormal development of the innervation of EOMs:**		
CCDD including CFEOM, Duane syndromes etc… (*mutations of genes implicated in the growth of oculomotor nerves e.g., kinesins, transcription factors, guidance of axons*)		
**Abnormal EOMs proprioception at the level of receptors**		
**Innate nerve palsies**		**Acquired nerve palsies**
**CENTRAL ORIGINS:**	Abnormal development of the visual paths(role of ephrins)	Accommodative or non-accommodative esotropia:
Concomitant strabismus (>90%)	– Abnormal routing of ganglion cell axons – Misrouting and abnormal retinotopy – Abnormal cortico-cortical connections	✓50% of cases in Caucasians ✓33% of cases in Asians
≪Congenital≫ strabismus	Abnormal development of neuronal activity in visual system	Exophoria/Exotropia
✓10% of cases	– Abnormal retinal waves – Abnormal visual perception – Abnormal balance excitation/inhibition – Abnormal synchronization of neural activity	✓50% of cases in Asians ✓33% of cases in Caucasians
	Abnormal development of neuronal activity in oculomotor system	
	– Abnormal extraocular proprioceptive inputs from EOMs to V1 – Abnormal activity of the vergence neurons and abnormal cortical control – Abnormal activity in Superior Colliculus Cerebellum and /or vestibular pathways	

Origins at left are those that subtend strabismus occurring before 8 postnatal (PN) months while origins designated on the right are those subtending strabismus occurring beyond 2 postnatal years. Those mentioned in the middle of the table might subtend both early and late strabismus. In most cases, except in cases of palsies, whether they are situated in periphery or centrally, all the abnormalities being mentioned here have likely a genetic origin, expressing at different periods after birth (see text and Table 2). EOMs, extraocular muscles; CCDD, Congenital cranial dysinnervation disorders; CFEOM, Congenital fibrosis of the extraocular muscles; V1, primary visual cortex. Concerning the epidemiology of strabismus, see for instance: Chia et al. (2007), Greenberg et al. (2007), Mohney (2007).

**Table 2:** **Genetics of strabismus**.

**PERIPHERAL**	
** •Genetic muscular diseases e.g.,**	
**Gene**	**Disease**
DMD gene (Xp21.2)	*Duchenne muscular dystrophy and Becker muscular dystrophy*
FRG1, ANT1 et DUX4 (4q35)	*Facio-scapulo-humeral muscular dystrophy*
DMPK (19q13-2)	*Steinert myotonic dystrop*
** •Genetic abnormal development of nerves e.g.,**	
**Gene**	**Disease**
KIF21A (12q12)	*CFEOM*
PHOX2A (11q13)	*CFEOM 2*
Gene located at 16qter	*CFEOM 3*
Genes located at 8q13 and 2q31	*Duane syndromes*
SALL4 (20q13)	*DRRS*
ROBO3 (11q23)	*HGPPS*
HOXA1 (7p15)	*BSAS and ABDS*
**CENTRAL**	
** • Genetic trait in accommodative esotropia and inheritance of refractive errors such as hyperopia**	
** • Genetic trait in exophoria/exotropia**	
Complex genetic in heritance, with the possible implication of recessive and dominant genes	

*This table allows summarizing already identified genes being responsible for strabismus. In the periphery, some specific genes can be associated to some specific diseases concerning the extraocular muscles or the oculomotor nerves. Centrally, genes are also implicated in generating strabismus but their identification is more difficult, in particular because not one single gene is implicated. In addition, they might be dominant or recessive. CFEOM, Congenital fibrosis of the extraocular muscles; DRRS, Duane Radial Ray Syndrome; HGPPS, Horizontal Gaze Palsy with Progressive Scoliosis; BSAS, Bosley-Salih-Alorainy Syndrome; ABDS, Athabascan Brainstem Dysgenesis Syndrome*.

By contrast, other forms of strabismus (mostly concomitant) are presently poorly understood while they are the most numerous (>90%). About 10% of them are “congenital” strabismus (thus occurring before 8 PN months), while the remaining occur at a later stage. In this latter situation, they are characterized by either an accommodative or non-accommodative esotropia or an exophoria or an exotropia (cf. Table 1). They are also known to have a genetic origin (see Table 2). However, by contrast to strabismus with an identified (peripheral) origin, those forms of strabismus are likely related to both recessive and dominant genes, thus resulting from a complex genetic inheritance. To move forward, we have proposed different possibilities to justify the emergence of such “uncharacterized” forms of strabismus, by evoking the occurrence of abnormal development of “central” paths and abnormal development of “central” neural activity. This evidently concerns both the visual and the oculomotor systems, up to the cortex, since they are closely related. Indeed, these forms of strabismus, with unknown origins, are generally “supposed” to have a central origin. Of interest, some of the anatomo-functional abnormalities we have proposed as being responsible for strabismus may take place before birth or postnatally, with consequences that the different forms of strabismus with a central origin may have early or late onset. Thus we did not dissociate them in Table 1. This may help to justify, however, the occurrence of early and late strabismus. It is not necessary to underline that many other mechanisms could have been proposed as the possibilities are vast. To explain each form of strabismus supposes that a lot of mechanisms might be at source of strabismus with a central origin. Most of the mechanisms we propose are clearly hypothetical and remain to be proven. But, as indicated above, some mechanisms are already supported by precise data. For example, disrupting callosal connections alters the alignment of the eyes (Elberger, 1979; Payne et al., 1981; Elberger and Hirsch, 1982). A split between the ventral and the dorsal streams such as the one occurring in Williams syndrome most often leads to strabismus (Atkinson et al., 2001). An abnormal visual perception from one eye may also lead to strabismus (e.g., Quick et al., 1989; Niechwiej-Szwedo et al., 2010). One may also consider that disturbances of synchrony in the developing brain, associated with aberrant neurodevelopment, may also be a source of strabismus, at least in some cases, because of the growing evidence that they generally subtend cognitive dysfunction (e.g., Uhlhaas and Singer, 2006; Uhlhaas et al., 2009a,b, 2011).

### About the origins of binocular vision loss

Our article also aimed at reconsidering the question of the origins of binocular vision loss, including 3D perception loss and acuity loss. Such deficits are evidently classic consequences of strabismus, because of the abnormal visual experience they generate postnatally. But we are also convinced that their respective origins are central, at least in some cases. Logical deductions lead to such a hypothesis. As outlined above, any abnormality within the visual network (because of abnormal retinal waves prenatally or otherwise) may lead to abnormal visual perception, with a central origin. Thus, for example, an abnormal segregation of inputs from both eyes and/or any abnormality in the organization of the retina will lead to the development of abnormal ocular dominance maps and/or abnormal retinotopic maps in visual cortex. This unavoidably leads to an abnormal binocular integration. Abnormalities in the orientation and/or the spatial frequency maps in V1 (or beyond) will also unavoidably lead to amblyopia, and hence to binocular vision loss. More generally, any alteration within the M, P, or K pathways, from retina to cortex, likely with a genetic origin, will lead to such alterations. Any abnormal synchronization of neural activity at the cortical (or sub-cortical) level will also lead to amblyopia and/or abnormal 3D perception. Of interest in the present context, each may additionally lead to strabismus (cf. text for details, Section abnormal visual perception may induce strabismus).

### How to improve treatment of strabismus and binocular vision loss in the future

Taking all the above developments into account, the main question is now: “How can ophthalmologists better assist strabismic patients and those with binocular vision loss in the future?” Evidently, they will have to continue applying the conventional treatments to limit the *consequences* of strabismus due to an abnormal postnatal experience. But considering what is included in the present article, more innovative treatments and new strategies would also need to be used, in particular with respect to the *origins* of strabismus. The same applies to binocular vision loss.

#### Improving treatment of strabismus in the future

***Treatments being used for strabismus***.

*Conventional treatments*. As summarized in Table 3, evidently, the main aim of the ophthalmologist is presently to eliminate or to limit perceptive abnormalities due to strabismus in order to recover visual acuity, rectitude of the eyes and normal binocular vision as much as possible. For that, classically, the ophthalmologist currently addresses these functional disorders during the critical period and intervenes in periphery, at the level of the eyes, through refractive treatment, amblyopia treatment, binocular treatment and/or surgical treatment: (a) Glasses are prescribed after cycloplegia, which allows the correction of ametropia in order to control the influence of refractive errors and accommodative excess or lack in case of strabismus; (b) The treatment of (monocular) amblyopia is the priority since plasticity during the postnatal period decreases progressively over time and therapeutic success depends on the timing of the treatment. Refractive treatment is the first step of amblyopia treatment and can be effective in mild anisometropic amblyopias. But it must be strongly emphasized that the treatment of amblyopia also requires patching of the sound eye. Some studies by the PEDIG, a group of North American Pediatric Ophthalmologists exploring different amblyopia therapies (Beck, 1998), have suggested that a “soft” treatment can be as efficacious as a “harder” one. For instance, 6 h patching vs. 2 h patching of the good eye would be sufficient (see, for example, Rees et al., 2007). However, it must be pointed out that only an improvement of vision was expected with such treatment, not a complete healing of amblyopia. To reach this latter stage full time patching is required for several weeks. Patching an eye increases the cortical input to the cortex from the amblyopic eye, and this effect is necessary to increase visual acuity. Note, however, that the efficacy of the “patchy method” greatly varies with the age of the patient since the plastic properties of the visual system evolve during the critical period (Epelbaum et al., 1993); (c) When visual acuity is recovered in strabismic patients, surgery allows the realignment of the eyes which is necessary to ensure binocular vision. Surgery has a direct effect on the EOMs, allowing the modification of the position of the eye: a recession of a muscle diminishes its effective force on the eye, whereas reinforcement is allowed by a muscular resection. However, this requires intervention on the EOMs, in particular at the level of their tendons where major muscle receptors are located. By taking into account that extraocular proprioception plays a major role in the maturation of V1, at least during the first half of the critical period, one must be aware that this may induce unfortunate consequences in the development of the brain (see above).

**Table 3:** **Main objectives and main strategies of the ophthalmologist in case of strabismus**.

**Main objectives of the opthalmologist in case of strabismus**	
Presently, the main aim of the ophthalmologist is to eliminate or to limit perceptive abnormalities due to strabismus in order to recover visual acuity, rectitude of the eyes and normal binocular vision as much as possible	In the future, the ophthalmologist will still have to eliminate or to limit perceptive abnormalities due to strabismus but will ALSO have to prevent strabismus and perceptive abnormalities to develop
**Main strategies that may be used to treat strabismus**	
**Treatments being used presently in order to treat the *consequences* of strabismus**	Potential strategies for the future
	➢ **To *better treat* the consequences of strabismus by:**
➢ **Conventional treatments:** –Refractive treatment: glasses, lenses, refractive surgery –Patches / temporary monocular deprivation –EOMs surgery	Combining more systematically newly developed strategies (e.g. binocular stimulations and TMS) in addition to the conventional treatments
	➢ **To *better understand* the origins and consequences of strabismus in patients by:** –Acquiring a better knowledge of the timing of the different phases of normal visual development in infancy (by relating tightly genes, molecular processes, anatomy, function) –Acquiring a better knowledge of the mechanisms leading to the alteration of the brain development –Using more systematically EEG and MEG recordings, in combination with psychophysical analysis –Combining fMRI and psychophysical analysis (if possible)
	Performing systematically a genetic screening
	➢ **To *anticipate* against negative effects of strabismus** –In patients that are susceptible to develop strabismus, diagnosed through genetic screening: perform treatments early (both conventional and newly developed ones) –Genetic therapy (when it will be possible)

*See text for details*.

*New treatments*. The “monocular patchy method” has been used to treat amblyopia since major findings by Hubel and Wiesel (1965). But, over time, it has been thought to reduce binocular stimulation to the visual system. This is a very important issue, since despite what has been thought for decades, neurons in visual cortex have finally been shown to remain binocular in spite of strabismus, even if binocular interactions are abnormal (see above). This is the reason why new strategies implicating binocular stimulations are presently being developed to treat amblyopia and binocular vision loss in strabismic (and anisometropic) subjects. Hess and his colleagues are among the most active in that field, with their strategy to suppress interocular suppression in order to recover acuity by the amblyopic eye and 3D perception (Baker et al., 2007; Mansouri et al., 2008; Hess et al., 2010a,b, 2011; Zhou et al., 2012 cf. also Hess et al., 2014 for review). For that, they have developed dichoptic devices that allow a binocular stimulation with different images in each eye, the combination of which is stereoscopic. Without going into detail, during these binocular stimulations, the image with the lowest contrast is presented to the fellow fixing eye, which allows enhanced performance in the other eye. The practical management of patients using this approach is still under development but this approach for amblyopia, binocular vision loss and, more generally, consequences of strabismus on visual perception appears quite promising, more especially as it is also able to be used in adulthood (e.g., To et al., 2011; Black et al., 2012; Li et al., 2013; see also Hess and Thompson, 2013 for review). During the treatment of amblyopia (either strabismic or anisometropic), therapy sessions of this type could be carried out, in addition to patching therapy, in order to improve the treatment of the effects of strabismus. Patching would increase the monocular input from one eye to the cortex, avoiding the asymmetry of signal until a balance is obtained, whereas the binocular treatment would avoid interocular suppression and would help to restore binocular function. In the particular case of strabismus, it would also require alignment of the eyes to avoid inducing diplopia. The necessary conventional approaches through refractive treatment, amblyopia treatment and surgical treatment will need to be combined with these new approaches.

Another new and promising strategy to recover visual function in strabismic (and anisometropic) adults is also under development. This time, it consists of non-invasive transcranial brain stimulations, with the aim of modifying the balance of excitation and inhibition in the visual cortex (Thompson et al., 2010; Clavagnier et al., 2013; Spiegel et al., 2013; see also Hess and Thompson, 2013 for review). Used alone or in combination with binocular therapy (cf. see above) it may assist in the treatment of both amblyopia and binocular vision loss (e.g., Spiegel and Li, 2013). A precise knowledge of the brain abnormalities that are present in each case of strabismus is required to adjust the brain stimulation temporally and spatially, i.e., performing the treatment at the correct time during development and on the correct region of the brain to treat the consequences of strabismus. In the future it could also be possible to reshape the brain and the abnormalities that are responsible for strabismus (abnormal synchrony, lack of activity, etc.).

Indeed, the two new methods we have presented only aim now at treating consequences of strabismus. To further advance, on the basis of reliable indices, one may imagine that such methods may also be developed to treat the origins of strabismus and to prevent strabismus to develop in the future (see above for justification).

***Potential future strategies to further improve treatment of strabismus, in particular with respect to its origin***. As early as possible, ophthalmologists might improve the current management of strabismus by targeting both the *consequences* AND the *origin(s)* of strabismus. As set out above, very promising strategies are already being developed to limit or even eliminate amblyopia and binocular vision loss due to strabismus (or anisometropy), including in adulthood. Here, we suggest additional potential strategies for the future (cf. Table 3 for summary). We propose first *to reach a better understanding* of both the origins and the consequences of strabismus than presently. Second, we are also convinced that to apply an *anticipating strategy* against strabismus, whenever possible, would be also very pertinent.

*To better understand the origins and consequences of strabismus*. Five strategies at least may be proposed to ophthalmologists in order to reach a better understanding of both the origins and the consequences of strabismus.

- **To acquire a better knowledge of the timing of the different phases of normal visual development in human**. Presently, ophthalmologists know a lot about the normal development of visual perception in humans, i.e., about “visual function” (cf. Figure 1). For example, they know perfectly that acuity increases from birth to reach its maximum between 4 and 8 PN years. They also know that binocular vision appears suddenly at about 3 PN months but continues to improve up to 9 PN years. But to move forward in the future, ophthalmologists would need to acquire a precise knowledge about the normal development of the visual system itself, from eye to cortex, before and after birth. This would constitute an additional reference for them. As illustrated above, this would require first the ability to relate genes, molecules, anatomy and neural activity to each “phase” of development of the visual system, including their timing. The same holds true concerning the timing of the critical periods of each attribute of the visual scene. As illustrated above, much is known already about all of these aspects in various mammal species, including monkeys, cats, ferrets, rats and mice. The general phases of development we are interested in here are basically the same in all mammals, except with respect to the *timings* (e.g., Huberman et al., 2008; Espinosa and Stryker, 2012 for review). Thus, finally, in relation to such an issue, what is missing principally for humans is the timing of the different phases of development. Second, ophthalmologists would have to understand how interactions between distributed brain regions mature with age to produce sophisticated cognitive functions such as visual perception (see Section Systematic Use of EEG and/or MEG and/or fMRI Recordings, in Combination with Psychological Analysis below for details). Interestingly, some recent reviews have started to treat these aspects by taking into account the time courses of neural proliferation, neural migration, apoptosis, synaptogenesis, establishment of neural circuits and myelination in the different regions of the human brain, including the visual cortex, before and after birth (e.g., Tau and Peterson, 2010; Menon, 2013). For that, neuroimaging, EEG and MEG recordings, together with traditional investigational approaches such as histological studies and cellular and molecular biology, have been used. Improving our understanding of these developmental processes in humans is likely a major key to the successful treatment of strabismus and binocular visual loss. To have this type of information with respect to the oculomotor system would be also very interesting.
- **To acquire a better knowledge of the mechanisms leading to the alteration of brain development in case of strabismus**. As illustrated in the present article, few data are finally available regarding the origins of early or late strabismus, in particular when these origins are central. We have proposed different mechanisms but most still need to be confirmed as real sources of strabismus. In contrast, much is already known about both the anatomical and functional consequences of strabismus, from retina to cortex (e.g., Wong, 2012 for review). But again, it would be interesting for ophthalmologists to know more about the mechanisms that are implicated during these plastic changes in the brain. Such information is necessary to develop new treatment strategies. As an example, if remaining binocularity of cortical neurons had not been shown to be sustained after strabismus (Chino et al., 1994; Sengpiel et al., 1994), it is possible that Hess and his colleagues would have not developed their promising binocular therapy (see above).
- **Systematic use of EEG and/or MEG and/or fMRI recordings, in combination with psychological analysis**. Among other possibilities, we have proposed here that any anatomo-functional abnormality within the visual cortex may lead to strabismus (and amblyopia and/or binocular vision loss) by altering synchrony. This is in line with increasing evidence that disturbances of synchrony in the developing brain are associated with aberrant neurodevelopment and subtend cognitive dysfunctions (Uhlhaas and Singer, 2006; Uhlhaas et al., 2009a,b, 2011). Interestingly, in order to help physicians and neurologists to establish a diagnostic in the future, various authors have now started to address the question of the relationship between neural synchrony and the underlying anatomical and physiological changes that occur during normal brain development (e.g., Menon, 2013 for review). They also aim at associating a specific disruption of dynamic processes to abnormal connectivity and specific disturbances of cognitive or executive functions (see above; see also Menon, 2013 for review). Their idea is that recording dynamics of brain activity using electroencephalography (EEG) and/or magnetoencephalography (MEG) with or without new psychophysical measurements of visual perception must somehow reflect the functional architecture of cortical networks. “Because this architecture is determined by genetic factors and modified by experience, spontaneous or evoked activity patterns should contain information about evolutionary and epigenetically acquired knowledge regarding the world and serve as a convert internal model for perception and action” (e.g., Singer, 2013 for review). This indicates that recording resting-state or evoked activity from the brain would be sufficient, at least in principle, to identify whether something is wrong in the cortical networks. As suggested by Buzsáki et al. (2013), “oscillopathies or dysrhythmias could reflect malfunctioning network and, as endophenotypes, could assist in specifying diagnostics.” This has not been applied as yet but it is a promising possibility for the future, in particular in the context in which we are interested here. One might speculate that to establish specific relations between “oscillopathies” or “dysrhythmias” in the brain and various types of strabismus might greatly help, both to prevent strabismus to develop and to cure dilatory consequences due to strabismus, at least in some cases. Such techniques are particularly interesting here in that they are non-invasive techniques and can be used in infants at the earliest ages (e.g., Csibra et al., 2000). The fMRI could also help in assessing abnormal connectivity, although it is not easy to perform this in young infants (e.g., Li et al., 2011).

*To anticipate against negative effects of strabismus*. The EEG and/or MEG and/or fMRI recordings, with or without psychological analysis, might be used for the diagnosis of brain “abnormalities” that are present in the case of strabismus, and that should be treated, for example by using TMS (see above). Also, it should be necessary to anticipate the negative effects of strabismus on the visual system, and one may propose to perform a genetic screening for that purpose. In the future, one may even consider repairing altered genes.

- **To perform systematically a genetic screening**. Genetic knowledge, whatever its complexity, may be used (see above). Gene profiles can now be established relatively easily in humans, using non-invasive methods. Some genes at least are already known to be associated with strabismus (see above). To identify more would certainly assist, whether they are implicated in strabismus or absence of binocular vision. Establishing, as early as possible after birth, that a child is at risk of developing strabismus by showing that he or she has one or more affected gene(s) would at least allow ophthalmologists to develop a *treatment plan* to limit dilatory consequences of strabismus. Indeed, as outlined above, consequences of strabismus and/or absence of binocular vision might be rather difficult to treat in particular when strabismus has occurred very early postnatally. Thus, to diagnose strabismus as early as possible would only be beneficial.
- **To use genetic therapy**. In the future, even if it is currently not a reality, one may also expect to use innovative technologies such as genetic therapy. It would allow the repair of altered genes susceptible to induce strabismus and/or loss of binocular vision, at least in some cases. Is it a utopia to be able to repair the brain itself in the present context? Whatever the answer, intervening as early as possible is the best strategy.

#### How to improve treatment of binocular vision loss in the future?

As mentioned above, some newly developed strategies such as binocular therapy and TMS stimulations are improving the treatment of binocular vision loss caused by strabismus (e.g., Hess and Thompson, 2013; Hess et al., 2014 for reviews). But it is important to emphasize that, at least presently, binocular vision cannot be restored whatever the form of strabismus. In particular, it cannot be obtained after an early onset strabismus. Congenital binocular vision loss, without strabismus, is also presently impossible to treat. In both cases, this is likely due to a major insult of the maturational process of the visual system with respect to binocularity, occurring either before or soon after birth, due to innate/genetic factors. Evidently, in these cases, neural networks in the brain would have to be “repaired” through either reshaping (if early enough after birth) or by activating functional sleepy synapses or otherwise. However, to reach such goals, the origin and the exact characteristics of the disease would need to be identified in great detail. Appropriate strategies to remove or even to prevent binocular vision loss should then be developed. Our suggestion for ophthalmologists is that they increase their knowledge concerning the different phases that characterize the normal development of the visual system.

Combining better knowledge of the origins of strabismus and loss of binocular vision with new therapies will no doubt allow the more efficient management of these pathologies.

### Conflict of interest statement

The authors declare that the research was conducted in the absence of any commercial or financial relationships that could be construed as a potential conflict of interest.

## References

[B1] Ackman J. B. Burbridge T. J. Crair M. C. (2012). Retinal waves coordinate patterned activity throughout the developing visual system. Nature 490, 219–225 10.1038/nature11529 23060192 PMC3962269

[B2] Ashton J. A. Boddy A. Dean S. R. Milleret C. Donaldson I. M. (1988). Afferent signals from cat extraocular muscles in the medial vestibular nucleus, the nucleus praepositus hypoglossi and adjacent brainstem structures. Neuroscience 26, 131–145 10.1016/0306-4522(88)90132-7 3419584

[B3] Ashton J. A. Milleret C. Donaldson I. M. (1989). Effects of afferent signals from the extraocular muscles upon units in the cerebellum, vestibular nuclear complex and oculomotor nucleus of the trout. Neuroscience 31, 529–541 10.1016/0306-4522(89)90395-3 2797449

[B4] Atkinson J. Anker S. Braddick O. Nokes L. Mason A. Braddick F. (2001). Visual and visuospatial development in young children with Williams syndrome. Dev. Med. Child Neurol. 43, 330–337 10.1017/S0012162201000615 11368486

[B5] Baker D. H. Meese T. S. Mansouri B. Hess R. F. (2007). Binocular summation of contrast remains intact in strabismic amblyopia. Invest. Ophthalmol. Vis. Sci. 48, 5332–5338 10.1167/iovs.07-0194 17962490

[B6] Barrett B. T. Bradley A. McGraw P. V. (2004). Understanding the neural basis of amblyopia. Neuroscientist 10, 106–117 10.1177/1073858403262153 15070485

[B7] Batini C. Buisseret P. (1974). Sensory peripheral pathway from extrinsic eye muscles. Arch. Ital. Biol. 112, 18–32 4827422

[B8] Batini C. Buisseret P. Buisseret-Delmas C. (1975). Trigeminal pathway of the extrinsic eye muscle afferents in cat. Brain Res. 85, 74–78 10.1016/0006-8993(75)91008-2 162843

[B9] Beck R. W. (1998). The pediatric eye disease investigator group. J. AAPOS 2, 255–256 10.1016/S1091-8531(98)90079-9 10646744

[B10] Berlucchi G. (2014). Visual interhemispheric communication and callosal connections of the occipital lobes. Cortex 56, 1–13 10.1016/j.cortex.2013.02.001 23489777

[B11] Beurdeley M. Spatazza J. Lee H. H. Sugiyama S. Bernard C. Di Nardo A. A. (2012). Otx2 binding to perineuronal nets persistently regulates plasticity in the mature visual cortex. J. Neurosci. 32, 9429–9437 10.1523/JNEUROSCI.0394-12.2012 22764251 PMC3419577

[B12] Birch E. E. (2013). Amblyopia and binocular vision. Prog. Retin. Eye Res. 33, 67–84 10.1016/j.preteyeres.2012.11.001 23201436 PMC3577063

[B13] Black J. M. Hess R. F. Cooperstock J. R. To L. Thompson B. (2012). The measurement and treatment of suppression in amblyopia. J. Vis. Exp. 70:e3927 10.3791/3927 23271400 PMC3575204

[B14] Brodsky M. C. (2011). Dissociated vertical divergence: cortical or subcortical in origin? Strabismus 19, 67–68 10.3109/09273972.2011.575434 21635170

[B15] Bucci M. P. Seassau M. (2012). Saccadic eye movements in children: a developmental study. Exp. Brain Res. 222, 21–30 10.1007/s00221-012-3192-7 22836522

[B16] Bucci M. P. Seassau M. (2014). Vertical saccades in children: a developmental study. Exp. Brain Res. 232, 927–934 10.1007/s00221-013-3805-9 24352609

[B17] Bui Quoc E. Ribot J. Quenech'du N. Doutremer S. Lebas N. Grantyn A. (2012). Asymmetrical interhemispheric connections develop in cat visual cortex after early unilateral convergent strabismus: anatomy, physiology, and mechanisms. Front. Neuroanat. 5:68 10.3389/fnana.2011.00068 22275883 PMC3257851

[B18] Buisseret P. Maffei L. (1977). Extraocular proprioceptive projections to the visual cortex. Exp. Brain Res. 28, 421–425 885187 10.1007/BF00235720

[B19] Buisseret P. Gary-Bobo E. Imbert M. (1978). Ocular motility and recovery of orientational properties of visual cortical neurons in dark-reared kittens. Nature 272, 816–817 10.1038/272816a0 643071

[B20] Buisseret P. Gary-Bobo E. Milleret C. (1988). Development of the kitten visual cortex depends on the relationship between the plane of eye movements and visual inputs. Exp. Brain Res. 72, 83–94 10.1007/BF00248503 3169198

[B21] Buisseret P. (1995). Influence of extraocular muscle proprioception on vision. Physiol. Rev. 75, 323–338 7724665 10.1152/physrev.1995.75.2.323

[B22] Buzsáki G. Logothetis N. Singer W. (2013). Scaling brain size, keeping timing: evolutionary preservation of brain rhythms. Neuron 80, 751–764 10.1016/j.neuron.2013.10.002 24183025 PMC4009705

[B23] Cang J. Kaneko M. Yamada J. Woods G. Stryker M. P. Feldheim D. A. (2005a). Ephrin-as guide the formation of functional maps in the visual cortex. neuron. Neuron 48, 577–589 10.1016/j.neuron.2005.10.026 16301175 PMC2424263

[B24] Cang J. Rentería R. C. Kaneko M. Liu X. Copenhagen D. R. Stryker M. P. (2005b). Development of precise maps in visual cortex requires patterned spontaneous activity in the retin. Neuron 48, 797–809 10.1016/j.neuron.2005.09.015 16337917 PMC2562716

[B25] Canturk S. Oto S. Kizilkilic O. Karaca S. Akova Y. A. (2008). Rhombencephalosynapsis associated with infantile strabismus. Strabismus 16, 23–27 10.1080/09273970701863610 18306119

[B26] Cardin J. A. Carlen M. Meletis K. Knoblich U. Zhang F. Deisseroth K. (2009). Driving fast-spiking cells induces gamma rhythm and controls sensory responses. Nature 459, 663–667 10.1038/nature08002 19396156 PMC3655711

[B27] Castelo-Branco M. Neuenschwander S. Singer W. (1998). Synchronization of visual responses between the cortex, lateral geniculate nucleaus, and retina in the anesthetized cat. J. Neurosci. 18, 6395–6410 9698331 10.1523/JNEUROSCI.18-16-06395.1998 PMC6793201

[B28] Chan S. T. Tang K. W. Lam K. C. Chan L. K. Mendola J. D. Kwong K. K. (2004). Neuroanatomy of adult strabismus: a voxel-based morphometric analysis of magnetic resonance structural scans. Neuroimage 22, 986–994 10.1016/j.neuroimage.2004.02.021 15193630

[B29] Chapman B. Stryker M. P. Bonhoeffer T. (1996). Development of orientation preference maps in ferret primary visual cortex. J. Neurosci. 16, 6443–6453 8815923 10.1523/JNEUROSCI.16-20-06443.1996 PMC2669086

[B30] Chavasse F. B. (1939). Worth's Squint or the Binocular Reflexes and the Treatment of Strabismus, 7th Edn. Philadelphia, PA: P. Blackiston's son

[B30a] Chia A. Roy L. Seenyen L. (2007). Comitant horizontal strabismus: an Asian perspective. Br. J. Ophthalmol. 91, 1337–1340 10.1136/bjo.2007.116905 17475715 PMC2000992

[B31] Chino Y. M. Shansky M. S. Jankowski W. L. Banser F. A. (1983). Effects of rearing kittens with convergent strabismus on development of receptive field properties in striate cortex neurons. J. Neurophysiol. 50, 265–286 6875648 10.1152/jn.1983.50.1.265

[B32] Chino Y. M. Smith E. L. 3rd. Yoshida Z. Cheng H. Hamamoto J. (1994). Binocular interactions in striate cortical neurons of cats reared with discordant visual inputs. J. Neurosci. 14, 5050–5067 8046467 10.1523/JNEUROSCI.14-08-05050.1994 PMC6577165

[B33] Clavagnier S. Thompson B. Hess R. F. (2013). Long lasting effects of daily theta burst rTMS sessions in the human amblyopic cortex. Brain Stimul. 6, 860–867 10.1016/j.brs.2013.04.002 23664756

[B34] Cooper S. Daniel P. M. (1949). Muscle spindles in human extrinsic eye muscles. Brain 72, 1–21 10.1093/brain/72.1.1 18151578

[B35] Cordones I. Gomez C. M. Escudero M. (2013). Cortical dynamics during the preparation of antisaccadic and prosaccadic eye movements in humans in a gap paradigm. PLoS ONE 8:e63751 10.1371/journal.pone.0063751 23671699 PMC3650078

[B36] Crair M. C. Gillespie D. C. Stryker M. P. (1998). The role of visual experience in the development of columns in cat visual cortex. Science 279, 566–570 10.1126/science.279.5350.566 9438851 PMC2453000

[B37] Csibra G. Davis G. Spratling M. W. Johnson M. H. (2000). Gamma oscillations and object processing in the infant brain. Science 290, 1582–1585 10.1126/science.290.5496.1582 11090357

[B38] Dell'Osso L. F. Wang Z. I. (2008). Extraocular proprioception and new treatments for infantile nystagmus syndrome. Prog. Brain Res. 171, 67–75 10.1016/S0079-6123(08)00610-9 18718284

[B39] Demer J. L. (2003). Ocular kinematics, vergence, and orbital mechanics. Strabismus 11, 49–57 10.1076/stra.11.1.49.14090 12789583

[B40] Demer J. L. Clark R. A. Crane B. T. Tian J. R. Narasimhan A. Karim S. (2008). Functional anatomy of the extraocular muscles during vergence. Prog. Brain Res. 171, 21–28 10.1016/S0079-6123(08)00604-3 18718278 PMC2881303

[B41] Donahue S. P. (2007). Clinical practice. Pediatric strabismus. N. Engl. J. Med. 356, 1040–1047 10.1056/NEJMcp051888 17347457

[B42] Donaldson I. M. L. (1979). Responses in cat suprasylvian cortex (Clare Bishop Area) to stretch of extraocular muscles. J. Physiol. 296, 60P–61P 529132

[B43] Donaldson I. M. L. Dixon R. A. (1980). Excitation of unist in the lateral geniculate and continguous nuclei of the cat by stretch of intrinsic ocular muscles. Exp. Brain Res. 38, 245–255 10.1007/BF00236643 6245900

[B44] Donaldson I. M. L. Long A. C. (1980). Interaction between extraocular proprioceptive of visual signals in the superior colliculus of the cat. J. Physiol. 298, 85–110 7359446 10.1113/jphysiol.1980.sp013069 PMC1279104

[B45] Donaldson I. M. L. (2000). The functions of the proprioceptors of the eye muscles. Philos. Trans. R. Soc. Lond. B Biol. Sci. 355, 1685–1754 10.1098/rstb.2000.0732 11205338 PMC1692902

[B46] Donders F. C. (1863). Zur Pathogenie des schielens. Albrecht von Graefes Arch Ophthalmol. 9, 99–154 10.1007/BF02720844

[B47] Donnelly U. M. (2012). Horizontal strabismus worldwide–what are the risk factors? Ophthalmic Epidemiol. 19, 117–119 10.3109/09286586.2012.681002 22568423

[B48] Duane A. (1869). A new classification of the motor anomalies of the eyes based upon physiological principles. Ann. Ophthalmol. Otolaryngol. 5, 969

[B49] Elberger A. J. (1979). The role of the corpus callosum in the development of interocular eye alignment and the organisation of the visual field in cat. Exp. Brain Res. 36, 71–85 10.1007/BF00238468 467536

[B50] Elberger A. J. Hirsch H. V. (1982). Divergent strabismus following neonatal callosal section is due to a failure of convergence. Brain Res. 239, 275–278 10.1016/0006-8993(82)90851-4 7093681

[B51] Engel A. K. König P. Kreiter A. K. Singer W. (1991). Stimulus-dependant neuronal oscillations in cat visual cortex: inter-columnar interactions as determined by cross-correlation analysis. Eur. J. Neurosci. 2, 588–606 10.1111/j.1460-9568.1990.tb00449.x 12106294

[B52] Engel A. K. Fries P. Singer W. (2001). Dynamic predictions: oscillations and synchrony in top-down processing. Nature Rev. 2, 704–716 10.1038/35094565 11584308

[B53] Engle E. C. (2006). The genetic basis of complex strabismus. Pediatr. Res. 59, 343–348 10.1203/01.pdr.0000200797.91630.08 16492969

[B54] Engle E. C. (2007). Genetic basis of congenital strabismus. Arch. Ophthalmol. 125, 189–195 10.1001/archopht.125.2.189 17296894

[B55] Epelbaum M. Milleret C. Buisseret P. Dufier J. L. (1993). The sensitive period for strabismic amblyopia in man. Ophthalmology 100, 323–327 10.1016/S0161-6420(13)32170-8 8460000

[B56] Espinosa J. S. Stryker M. P. (2012). Development and plasticity of the primary visual cortex. Neuron 75, 230–249 10.1016/j.neuron.2012.06.009 22841309 PMC3612584

[B57] Fillenz M. (1955). Responses in the brainstem of the cat to stretch of extrinsic ocular muscles. J. Physiol. 128, 182–199 14368582 10.1113/jphysiol.1955.sp005298 PMC1365762

[B58] Frégnac Y. Imbert M. (1978). Early development of visual cortical cells in normal and dark-reared kittens: relationship between orientation selectivity and ocular dominance. J. Physiol. (Lond.) 278, 27–44 671298 10.1113/jphysiol.1978.sp012290 PMC1282335

[B59] Fries P. Schröder J. H. Roelfsema P. R. Singer W. Engel A. K. (2002). Oscillatory neuronal synchronization in primary visual cortex as a correlate of stimulus selection. J. Neurosci. 22, 3739–3754 11978850 10.1523/JNEUROSCI.22-09-03739.2002 PMC6758402

[B60] Fries P. (2005). A mechanism for cognitive dynamics: neuronal communication through neuronal coherence. Trends Cogn. Sci. 9, 474–480 10.1016/j.tics.2005.08.011 16150631

[B61] Galli L. Maffei L. (1988). Spontaneous impulse activity of rat retinal ganglion cells in prenatal life. Science 242, 90–91 10.1126/science.3175637 3175637

[B62] Ghosh A. Antonini A. McConnell S. K. Shatz C. J. (1980). Requirement for subplate neurons in the formation of thalamocortical connections. Nature 347, 179–181 10.1038/347179a0 2395469

[B63] Graeber C. P. Hunter D. G. Engle E. C. (2013). The genetic basis of incomitant strabismus: consolidation of the current knowledge of the genetic foundations of disease. Semin. Ophthalmol. 28, 427–437 10.3109/08820538.2013.825288 24138051 PMC4098966

[B64] Gray C. M. Koenig P. Engel A. K. Singer W. (1989). Oscillatory responses in cat visual cortex exhibit inter-columner synchronization which reflects global stimulus properties. Nature 23, 334–337 10.1038/338334a0 2922061

[B64a] Greenberg A. E. Mohney B. G. Diehl N. N. Burke J. P. (2007). Incidence and types of childhood esotropia: a population-based study. Ophthalmology 114, 170–174 10.1016/j.ophtha.2006.05.072 17070595

[B65] Gregoriou G. G. Gotts S. J. Desimone R. (2012). Cell-type-specific synchronization of neural activity in FEF with V4 during attention. Neuron 73, 581–594 10.1016/j.neuron.2011.12.019 22325208 PMC3297082

[B66] Harauzov A. Spolidoro M. DiCristo G. De Pasquale R. Cancedda L. Pizzorusso T. (2010). Reducing intraocular inhibition in the adult visual cortex promotes ocular dominance plasticity. J. Neurosci. 6, 361–371 10.1523/JNEUROSCI.2233-09.2010 20053917 PMC6632513

[B67] Hess R. F. Mansouri B. Thompson B. (2010a). A new binocular approach to the treatment of amblyopia in adults well beyond the critical period of visual development. Restor. Neurol. Neurosci. 28, 793–802 10.3233/RNN-2010-0550 21209494

[B68] Hess R. F. Mansouri B. Thompson B. (2010b). A binocular approach to treating amblyopia: antisuppression therapy. Optom. Vis. Sci. 87, 697–704 10.1097/OPX.0b013e3181ea18e9 20622704

[B69] Hess R. F. Mansouri B. Thompson B. (2011). Restoration of binocular vision in amblyopia. Strabismus 19, 110–118 10.3109/09273972.2011.600418 21870914

[B70] Hess R. F. Thompson B. (2013). New insights into amblyopia: binocular therapy and noninvasive brain stimulation. J. AAPOS 17, 89–93 10.1016/j.jaapos.2012.10.018 23352385

[B71] Hess R. F. Thompson B. Baker D. H. (2014). Binocular vision in amblyopia: structure, suppression and plasticity. Ophthalmic Physiol. Opt. 34, 146–162 10.1111/opo.12123 24588532

[B72] Hipp J. F. Engel A. K. Siegel M. (2011). Oscillatory synchronization in large-scale cortical networks predicst perception. Neuron 69, 387–396 10.1016/j.neuron.2010.12.027 21262474

[B73] Huang P. C. Li J. Deng D. Yu M. Hess R. F. (2012). Temporal synchrony deficits in amblyopia. Invest. Ophthal. Vis. Sci. 53, 8325–8332 10.1167/iovs.12-10835 23139268

[B74] Hubel D. H. Wiesel T. N. (1965). Binocular interaction in striate cortex of kittens reared with artificial squint. J. Neurophysiol. 28, 1041–1059 5883731 10.1152/jn.1965.28.6.1041

[B75] Hubel D. H. Wiesel T. N. (1970). The period of susceptibility to the physiological effects of unilateral eye closure in kittens. J. Physiol. 206, 419–436 5498493 10.1113/jphysiol.1970.sp009022 PMC1348655

[B76] Huberman A. D. Feller M. B. Chapman B. (2008). Mechanisms underlying development of visual maps and receptive fields. Annu. Rev. Neurosci. 31, 479–509 10.1146/annurev.neuro.31.060407.125533 18558864 PMC2655105

[B77] Ingster-Moati I. Vaivre-Douret L. Bui Quoc E. Albuisson E. Dufier J. L. Golse B. (2009). Vertical and horizontal smooth pursuit eye movements in children: a neuro-developmental study. Eur. J. Paediatr. Neurol. 13, 362–366 10.1016/j.ejpn.2008.07.003 18799334

[B78] Innocenti G. M. Frost D. O. (1979). Effects of visual experience on the maturation of the efferent system to the corpus callosum. Nature 280, 231–234 10.1038/280231a0 450139

[B79] Keskinbora K. H. (2008). Ocular and oculomotor findings of Joubert syndrome. J. Pediatr. Ophthalmol. Strabismus 45, 5–6 10.3928/01913913-20080101-15 18286952

[B80] Kilner J. M. Baker S. N. Salenius S. Hari R. Lemon R. N. (2000). Human cortical muscle coherence is directly related to specific motor parameters. J. Neurosci. 20, 8838–8845 11102492 10.1523/JNEUROSCI.20-23-08838.2000 PMC6773054

[B81] Krauzlis R. J. Lovejoy L. P. Zénon A. (2013). Superior colliculus and visual spatial attention. Annu. Rev. Neurosci. 36, 165–182 10.1146/annurev-neuro-062012-170249 23682659 PMC3820016

[B82] Lennerstrand G. (2007). Strabismus and eye muscle function. Acta Ophthalmol. Scand. 85, 711–723 10.1111/j.1600-0420.2007.00853.x 17944625

[B83] Lewis R. F. Zee D. S. Hayman M. R. Tamargo R. J. (2001). Oculomotor function in the rhesus monkey after deafferentation of the extraocular muscles. Exp. Brain Res. 141, 349–358 10.1007/s002210100876 11715079

[B84] Lewis D. A. Hashimoto T. Volk D. W. (2005). Cortical inhibitory neurons and schizophrenia. Nat. Rev. Neurosci. 6, 312–324 10.1038/nrn1648 15803162

[B85] Li J. Shen C. (2001). Histological and ultrastructural studies of extraocular muscle proprioceptor in concomitant strabismus. Zhonghua Yan Ke Za Zhi 37, 200–202 11864422

[B86] Li X. Mullen K. T. Thompson B. Hess R. F. (2011). Effective connectivity anomalies in human amblyopia. Neuroimage 54, 505–516 10.1016/j.neuroimage.2010.07.053 20682351

[B87] Li X. Thompson B. Deng D. Chan L. Y. L. Yu M. Hess R. F. (2013). Dichoptic training enables the adult amblyopic brain to learn. Curr. Biol. 23, R308–R309 10.1016/j.cub.2013.01.059 23618662

[B88] Löwel S. Singer W. (1992). Selection of intrinsic horizontal connections in the visual cortex by correlated neuronal activity. Science 255, 209–212 10.1126/science.1372754 1372754

[B89] Löwel S. Schmidt K. E. Kim D. C. Wolf F. Hoffsüller F. Singer W. (1998). The layout of orientation and ocular dominance domains in area 17 of strabismic cats. Eur. J. Neurosci. 10, 2629–2643 10.1046/j.1460-9568.1998.00274.x 9767393

[B90] Lund R. D. Mitchell D. E. (1979). Asymmetry in the visual callosal connections of strabismic cats. Brain Res. 167, 176–179 10.1016/0006-8993(79)90274-9 455064

[B91] Mansouri B. Thompson B. Hess R. F. (2008). Measurement of suprathreshold binocular interactions in amblyopia. Vis. Res. 48, 2775–2784 10.1016/j.visres.2008.09.002 18809424

[B92] Mansouri B. Hansen B. C. Hess R. F. (2009). Disrupted retinotopic maps in amblyopia. Invest. Ophtalmol. Vis. Sci. 50, 3218–3225 10.1167/iovs.08-2914 19255157

[B93] Marillat V. Sabatier C. Failli V. Matsunaga E. Sotelo C. Tessier-Lavigne M. (2004). The slit receptor Rig-1/Robo3 controls midline crossing by hindbrain precerebellar neurons and axons. Neuron 43, 69–79 10.1016/j.neuron.2004.06.018 15233918

[B94] Mays L. E. (1984). Neural control of vergence eye movements: convergence and divergence neurons in midbrain. J. Neurophysiol. 51, 1091–1108 6726313 10.1152/jn.1984.51.5.1091

[B95] McConnell S. K. Ghosh A. Shatz C. J. (1989). Subplate neurons pioneer the first axon pathway from the cerebral cortex. Science 245: 978–982 10.1126/science.2475909 2475909

[B96] Menon V. (2013). Developmental pathways to functional brain networks: emerging principles. Trends Cogn. Sci. 17, 627–640 10.1016/j.tics.2013.09.015 24183779

[B97] Milleret C. Gary-Bobo E. Buisseret P. (1987). Réponses des neurones du cortex visuel (Aire 18) aux stimulations proprioceptives extraoculaires: évolution chez le chat normal ou élevé à l'obscurité et interactions avec l'activité visuelle. C. R. Acad. Sci. III 305, 531–536 3121143

[B98] Milleret C. Gary-Bobo E. Buisseret P. (1988). Comparative development of cell properties in cortical Area 18 of normal and dark-reared kittens. Exp. Brain Res. 71, 8–20 10.1007/BF00247518 3416960

[B99] Milleret C. Houzel J. C. Buser P. (1994). Pattern of development of the callosal transfer of visual information to cortical areas 17 and 18 in the normally-reared cat. Eur. J. Neurosci. 6, 193–202 10.1111/j.1460-9568.1994.tb00261.x 8167841

[B100] Milleret C. (1994). Visual callosal connections and strabismus. Behav. Brain Res. 64, 85–95 10.1016/0166-4328(94)90121-X 7840895

[B101] Milleret C. Houzel J. C. (2001). Visual interhemispheric transfer to areas 17 and 18 in cats with convergent strabismus. Eur. J. Neurosci. 13, 37–52 10.1046/j.1460-9568.2001.01360.x 11135012

[B101a] Mohney B. G. (2007). Common forms of childhood strabismus in an incidence cohort. Am. J. Ophthalmol. 144, 465–467 10.1016/j.ajo.2007.06.011 17765436

[B102] Montero V. M. Guillery R. W. (1978). Abnormalities of the cortico-geniculate pathway in Siamese cats. J. Comp. Neurol. 179, 1–12 10.1002/cne.901790102 8980714

[B103] Neuenschwander S. Singer W. (1996). Long-range synchronization of oscillatory light responses in the cat retina and lateral geniculate nucleus. Nature 379, 728–732 10.1038/379728a0 8602219

[B104] Niechwiej-Szwedo E. Goltz H. C. Chandrakumar M. Hirji Z. A. Wong A. M. (2010). Effects of anisometropic amblyopia on visuomotor behavior, I: saccadic eye movements. Invest. Ophthalmol. Vis. Sci. 51, 6348–6354 10.1167/iovs.10-5882 20671288 PMC5142839

[B105] Nyffeler T. Wurtz P. Lüscher H. R. Hess C. W. Senn W. Pflugshaupt T. (2006). Extending lifetime of plastic changes in the human brain. Eur. J. Neurosci. 24, 2961–2966 10.1111/j.1460-9568.2006.05154.x 17156218

[B106] Pasik P. Pasik T. Valciukas J. A. Bender M. B. (1971). Vertical optokinetic nystagmus in the split-brain monkey. Exp. Neurol. 30, 162–171 10.1016/0014-4886(71)90230-5 5542197

[B107] Payne B. R. (1990). Function of the corpus callosum in the representation of the visual field in cat visual cortex. Vis. Neurosci. 5, 205–211 10.1017/S0952523800000225 2278945

[B108] Payne B. R. (1991). Visual-field in the transcallosal sending zone of area 17 in the cat. Vis. Neurosci. 7, 201–219 10.1017/S095252380000403X 1721530

[B109] Payne B. R. Siwek D. F. (1991a). The visual map in the corpus callosum of the cat. Cereb. Cortex 1, 173–188 10.1093/cercor/1.2.173 1822731

[B110] Payne B. R. Siwek D. F. (1991b). Visual-field map in the callosal recipient zone at the border between areas 17 and 18 in the cat. Vis. Neurosci. 7, 221–236 10.1017/S0952523800004041 1721531

[B111] Payne B. R. Berman N. Murphy E. H. (1981). A quantitative assessment of eye alignment in cats after corpus callosum transection. Exp. Brain Res. 43, 371–376 7262229 10.1007/BF00238379

[B112] Péchereau A. (2013). Strabisme. Rapport de la Société Française d'Ophtalmologie. Paris: Elsevier Masson

[B113] Petros T. J. Shrestha B. R. Mason C. (2009). Specificity and sufficiency of EphB1 in driving the ipsilateral retinal projection. J. Neurosci. 29, 3463–3474 10.1523/JNEUROSCI.5655-08.2009 19295152 PMC2725437

[B114] Popple A. V. Levi D. M. (2005). Location coding by the human visual system: multiple topological adaptations in a case of strabismic amblyopia. Perception 34, 87–107 10.1068/p5345 15773609

[B115] Quick M. W. Tigges M. Gammon J. A. Boothe R. G. (1989). Early abnormal visual experience induces strabismus in infant monkeys. Invest. Ophthalmol. Vis. Sci. 30, 1012–1017 2722437

[B116] Rees M. G. Woo C. L. Optom B. (2007). Pediatric eye disease investigator group amblyopia treatment review. Am. Orthopt. J. 57, 99–103 10.3368/aoj.57.1.99 21149162

[B117] Richmond F. J. R. Johnson W. S. W. Baker R. S. Steinbach M. J. (1984). Palisade endings in human extraocular muscles. Invest. Ophthal. Vis. Sci. 25, 471–476 6706509

[B118] Rochefort N. L. Garaschuk O. Milos R. I. Narushima M. Marandi N. Pichler B. (2009). Sparsification of neuronal activity in the visual cortex at eye-opening. Proc. Natl. Acad. Sci. U.S.A. 106, 15049–15054 10.1073/pnas.0907660106 19706480 PMC2736444

[B119] Roelfsema P. R. König P. Engel A. K. Sireteanu R. Singer W. (1994). Reduced synchronization in the visual cortex of cats with strabismic amblyopia. Eur. J. Neurosci. 6, 1645–1655 10.1111/j.1460-9568.1994.tb00556.x 7874303

[B120] Sanfilippo P. G. Hammond C. J. Staffieri S. E. Kearns L. S. Melissa Liew S. H. Barbour J. M. (2012). Heritability of strabismus: genetic influence is specific to eso-deviation and independent of refractive error. Twin Res. Hum. Genet. 15, 624–630 10.1017/thg.2012.22 22877876

[B121] Schlenker M. Mirabella G. Goltz H. C. Kessler P. Blakeman A. W. Wong A. M. (2009). The linear vestibulo-ocular reflex in patients with skew deviation. Invest. Ophthalmol. Vis. Sci. 50, 168–174 10.1167/iovs.08-2254 18775861 PMC5104545

[B122] Schmidt K. E. Löwel S. (2008). Strabismus modifies intrinsic and inter-areal connections in cat area 18. Neuroscience 152, 128–137 10.1016/j.neuroscience.2007.08.038 18248913

[B123] Schmidt K. E. Kim D. S. Singer W. Bonhoeffer T. Löwel S. (1997). Functional specificity of long-range intrinsic and interhemispheric connections in the visual cortex of strabismic cats. J. Neurosci. 17, 5480–5492 9204930 10.1523/JNEUROSCI.17-14-05480.1997 PMC6793806

[B124] Schmidt K. E. Singer W. Galuske R. A. W. (2004). Processing deficits in primary visual cortex of amblyopic cats. J. Neurophysiol. 91, 1661–1671 10.1152/jn.00878.2003 14668297

[B125] Scholl B. Tan A. Y. Priebe N. J. (2013). Strabismus disrupts binocular synaptic integration in primary visual cortex. J. Neurosci. 33, 17108–17122 10.1523/JNEUROSCI.1831-13.2013 24155315 PMC3807032

[B126] Seeger M. Tear G. Ferres-Marco D. Goodman C. S. (1993). Mutations affecting growth cone guidance in Drosophila: genes necessary for guidance toward or away from the midline. Neuron 10, 409–426 10.1016/0896-6273(93)90330-T 8461134

[B127] Sengpiel F. Blakemore C. Kind P. C. Harrad R. (1994). Interocular suppression in the visual cortex of strabismic cats. J. Neurosci. 14, 6855–6871 7965083 10.1523/JNEUROSCI.14-11-06855.1994 PMC6577231

[B128] Sengpiel F. Jirmann K. U. Vorobyov V. Eysel U. T. (2006). Strabismic suppression is mediated by inhibitory interactions in the primary visual cortex. Cereb. Cortex 16, 1750–1758 10.1093/cercor/bhj110 16400161

[B129] Shatz C. J. Levay S. (1979). Siamese cat: altered connections of visual cortex. Science 204, 328–330 10.1126/science.432647 432647

[B130] Sherrington C. S. (1918). Observations on the sensual role of the proprioceptive nerve supply of the extrinsic ocular muscles. Brain 41, 332–343 10.1093/brain/41.3-4.332

[B131] Shieh P. B. (2013). Muscular dystrophies and other genetic myopathies. Neurol. Clin. 31, 1009–1029 10.1016/j.ncl.2013.04.004 24176421

[B132] Singer W. Rauschecker J. Von Gruenau M. (1979). Squint effects striate cortex cells encoding horizontal image movements. Brain Res. 170, 182–186 10.1016/0006-8993(79)90951-X 466400

[B133] Singer W. Gray C. M. (1995). Visual feature integration and the temporal correlation hypothesis. Annu. Rev. Neurosci. 18, 555–586 10.1146/annurev.ne.18.030195.003011 7605074

[B134] Singer W. (1999). Neuronal synchrony: a versatile code of the definition of relations. Neuron 24, 49–65 10.1016/S0896-6273(00)80821-1 10677026

[B135] Singer W. (2013). Cortical dynamics revisited. Trends Congn. Sci. 17, 616–626 10.1016/j.tics.2013.09.006 24139950

[B136] Sireteanu R. (2000). The binocular visual system in amblyopia. Strabismus 8, 39–51 10.1076/0927-3972(200003)811-6FT039 10970158

[B137] Smith S. L. Trachtenberg J. T. (2007). Experience-dependent binocular competition in the visual cortex begins at eye opening. Nat. Neurosci. 10, 370–375 10.1038/nn1844 17293862

[B138] Sohal V. S. Zhang F. Yishar O. Deisseroth K. (2009). Parvalbumin neurons and gamma rhythms enhace cortical circuit performance. Nature 459, 698–702 10.1038/nature07991 19396159 PMC3969859

[B139] Spiegel D. P. Li J. (2013). Transcranial direct current stimulation enhances recovery of stereopsis in adults with amblyopia. Neurotherapeutics 10, 831–839 10.1007/s13311-013-0200-y 23857313 PMC3805870

[B140] Spiegel D. P. Byblow W. D. Hess R. F. Thompson B. (2013). Anodal transcranial direct current stimulation transiently improves contrast sensitivity and normalizes visual cortex activation in individuals with amblyopia. Neurorehabil. Neural Repair 27, 760–769 10.1177/1545968313491006 23774122

[B141] Sugiyama S. Di Nardo A. A. Aizawa S. Matsuo I. Volovitch M. Prochiantz A. (2008). Experience-dependent transfer of Otx2 homeoprotein into the visual cortex activates postnatal plasticity. Cell 134, 508–520 10.1016/j.cell.2008.05.054 18692473

[B142] Tani T. Ribot J. O'Hashi K. Tanaka S. (2012). Parallel development of orientation maps and spatial frequency selectivity in cat visual cortex. Eur. J. Neurosci. 35, 44–55 10.1111/j.1460-9568.2011.07954.x 22211742

[B143] Tau G. Z. Peterson B. S. (2010). Normal development of brain circuits. Neuropsychopharmacology 35, 147–168 10.1038/npp.2009.115 19794405 PMC3055433

[B144] Ten Tusscher M. P. (2011). A neural model for cyclovertical eye movements and their disorders. Strabismus 19, 162–165 10.3109/09273972.2011.626833 22107122

[B145] Thompson B. Mansouri B. Koski L. Hess R. F. (2010). From motor cortex to visual cortex: application of noninvasive brain stimulation to amblyopia. Dev. Psychobiol. 54, 263–273 10.1002/dev.20509 22415915

[B146] To L. Thompson B. Blum J. R. Maehara G. Hess R. F. Cooperstock J. R. (2011). A game platform for treatment of amblyopia. IEEE Trans. Neural Syst. Rehabil. Eng. 19, 280–289 10.1109/TNSRE.2011.2115255 21335317

[B147] Tootell R. B. Hadjikhani N. K. Mendola J. D. Marrett S. Dale A. M. (1998). From retinotopy to recognition: fMRI in human visual cortex. Trends Cogn. Sci. 2, 174–183 10.1016/S1364-6613(98)01171-1 21227152

[B148] Torp-Pedersen T. Boyd H. A. Poulsen G. Haargaard B. Wohlfahrt J. Holmes J. M. (2010). Perinatal risk factors for strabismus. Int. J. Epidemiol. 39, 1229–1239 10.1093/ije/dyq092 20525734

[B149] Tusa R. J. Ungerleider L. G. (1988). Fiber pathways of cortical areas mediating smooth pursuit eye movements in monkeys. Ann. Neurol. 23, 174–183 10.1002/ana.410230211 3288083

[B150] Tychsen L. (2005). Can ophthalmologists repair the brain in infantile esotropia? Early surgery, stereopsis, monofixation syndrome, and the legacy of Marshall Parks. J. AAPOS 9, 510–521 10.1016/j.jaapos.2005.06.007 16414515

[B151] Uhlhaas P. J. Singer W. (2006). Neural synchrony in brain disorders: relevance for cognitive dysfunctions and pathophysiology. Neuron 52, 155–168 10.1016/j.neuron.2006.09.020 17015233

[B152] Uhlhaas P. J. Roux F. Singer W. Haenschel C. Sireteanu R. (2009a). The development of neural synchrony reflects late maturation and restructuring of functional networks in human. Proc. Natl. Acad. Sci. U.S.A. 106, 9866–9871 10.1073/pnas.0900390106 19478071 PMC2687997

[B153] Uhlhaas P. J. Roux F. Rodriguez E. Rotarska-Jagiela A. Singer W. (2009b). Neural synchrony and the development of cortical networks. Trends Cogn. Sci. 14, 72–80 10.1016/j.tics.2009.12.002 20080054

[B154] Uhlhaas P. J. Pipa G. Neuenschwander S. Wibral M. Singer W. (2011). A new look at gamma? (>60 Hz) γ-band activity in cortical networks: function, mechanisms and impairment. Prog. Biophys. Mol. Biol. 105, 14–28 10.1016/j.pbiomolbio.2010.10.004 21034768

[B155] Von Noorden G. K. (1978). Application of basic research data to clinical amblyopia. Ophthalmology 85: 496–504 10.1016/S0161-6420(78)35652-9 97608

[B156] Von Graefe A. (1854). Beitrage zur Physiologie und Pathologie der schiefen Augenmuskeln. Albrecht Von Graefes Arch. Ophthalmol. 1, 1–82 10.1007/BF02720619

[B157] Von Helmholtz H. (1866). Handbuch der physiologischen Optik. Voss, Leipzig, 1866; english translation: ‘Helmoltz’s Treatise on Physiological Optics', in Treatise on Physiologoical Optics, 3rd Edn. 1925, Vol. 3, eds Southall J. P. C. (Trans. Southall J. P. C.) (New York, NY: Dover; Optical Society of America).

[B158] Wang X. Cui D. Zheng L. Yang X. Yang H. Zeng J. (2012). Combination of blood oxygen level-dependent functional magnetic resonance imaging and visual evoked potential recordings for abnormal visual cortex in two types of amblyopia. Mol. Vis. 18, 909–919 22539870 PMC3335782

[B159] White L. E. Fitzpatrick D. (2007). Vision and cortical map development. Neuron 56, 327–338 10.1016/j.neuron.2007.10.011 17964249

[B160] Wong A. M. (2000). Anomalous retinal correspondence: neuroanatomic mechanism in strabismic monkeys and clinical findings in strabismic children. J. AAPOS 4, 168–174 10.1016/S1091-8531(00)70008-5 10849394

[B161] Wong A. M. (2012). New concepts concerning the neural mechanisms of amblyopia and their clinical implications. Can. J. Ophthalmol. 47, 399–409 10.1016/j.jcjo.2012.05.002 23036539

[B162] Worth C. (1915). Squint: its Causes, Pathology and Treatment, 4th Edn. London: John Bale and Danielson

[B163] Xu H. P. Furman M. Mineur Y. S. Chen H. King S. L. Zenisek D. (2011). An instructive role of patterned spontaneous retinal activity in mouse visual maps development. Neuron 70, 1115–1127 10.1016/j.neuron.2011.04.028 21689598 PMC3119851

[B164] Zernicki B. Stasiak M. Doty R. W. (1997). Habituation of ocular following reflex requires corpus callosum for interhemispheric transfer. Behav. Brain Res. 84: 269–274 10.1016/S0166-4328(97)83334-7 9079791

[B165] Ziakas N. G. Woodruff G. Smith L. K. Thompson J. R. (2002). A study of heredity as a risk factor in strabismus. Eye (Lond.) 16, 519–521 10.1038/sj.eye.6700138 12194061

[B166] Zhou J. Jia W. Huang C. B. Hess R. F. (2012). The effects of unilateral mean luminance on binocular combination in normal and amblyopic vision. Sci. Rep. 3, 1–7 10.1038/srep02012 23774670 PMC3684813

